# Fuzzy species borders of glacial survivalists in the Carpathian biodiversity hotspot revealed using a multimarker approach

**DOI:** 10.1038/s41598-021-00320-8

**Published:** 2021-11-03

**Authors:** Tomasz Mamos, Krzysztof Jażdżewski, Zuzana Čiamporová-Zaťovičová, Fedor Čiampor, Michał Grabowski

**Affiliations:** 1 Department of Invertebrate Zoology and Hydrobiology, Faculty of Biology and Environmental Protection, University of Lodz, Banacha 12/16, 90-237 Lodz, Poland; 2 ZooLab, Plant Science and Biodiversity Centre, Slovak Academy of Sciences, Dúbravská cesta 9, 845 23 Bratislava, Slovakia; 3 Department of Ecology, Faculty of Natural Sciences, Comenius University in Bratislava, Ilkovičova 6, 842 15 Bratislava, Slovakia

**Keywords:** Freshwater ecology, Population dynamics, Phylogenetics, Population genetics, Taxonomy

## Abstract

The Carpathians are one of the key biodiversity hotspots in Europe. The mountain chain uplifted during Alpine orogenesis and is characterised by a complex geological history. Its current biodiversity was highly influenced by Pleistocene glaciations. The goal of the current study was to examine the phylogenetic and demographic history of *Gammarus balcanicus* species complex in the Carpathians using multiple markers as well as to delimit, using an integrative approach, and describe new species hidden so far under the name *G. balcanicus*. Results showed that divergence of the studied lineages reaches back to the Miocene, which supports the hypothesis of their survival in multiple micro refugia. Moreover, the increase of their diversification rate in the Pleistocene suggests that glaciation was the driving force of their speciation. The climatic changes during and after the Pleistocene also played a major role in the demography of the local Carpathian lineages. Comparison of diversity patterns and phylogenetic relationships of both, the mitochondrial and nuclear markers, provide evidence of putative hybridisation and retention of ancient polymorphism (i.e., incomplete lineage sorting). The morphological examination supported the existence of two morphological types; one we describe as a *G. stasiuki* sp. nov. and another we redescribe as a *G. tatrensis* (S. Karaman, 1931).

## Introduction

European freshwaters are inhabited by numerous amphipod species classified in the genus *Gammarus* Fabricius, 1775*.* Extensive studies upon the diversity and taxonomy of this speciose genus in Europe were undertaken in the early twentieth century (e.g.,^1–4^). Subsequently, although 30 years passed since then, these efforts were summarized and critically revised in a series of works by G. Karaman and Pinkster^5–8^, with the result of the revision synonymizing most of the previously described species. Since that time, however, more than a dozen of new species were described from inland waters of Europe and adjacent regions (e.g.,^9–16^). Furthermore, during the recent decade, phylogenetic studies based on extensive molecular data sets have entirely altered traditional views on the taxonomy of the family Gammaridae and also on the definition and validity of genera belonging to this family^17–20^. Also, recent phylogeographic studies on several geographically widespread *Gammarus* morphospecies pointed out to outstanding cryptic and pseudo-cryptic diversity with an ancient divergence between phylogenetic lineages (e.g.,^14,15,20–25^). Unrevealing such a high level of hidden diversity stimulated further taxonomic studies, resulting in the description of new taxa, based either on a modern integrative approach using morphological, ultrastructural and molecular characters or, in the case of species for which the morphological distinction was impossible, based on molecular descriptive characters only (e.g.,^14,15^). However, the latter approach has been disputed for some years. Although it has several shortcomings (for discussion see^26^), it has been appreciated as a tool that helps to overcome the evident global taxonomic impediment (e.g.,^27,28^). Although the hypotheses of the species described in this way are viewed as interim, particularly if they are based only on a single molecular marker, they are as valid as those founded on morphological features. Such hypotheses can subsequently be verified using a multiproxy integrative approach^28^. Importantly, nowadays they may be used in broadly defined nature conservation, e.g., for fast, effective and precise quantification of biodiversity and detection of endemic lineages in the presumed diversity and endemism hotspots^25^.

The Carpathian Arc is the third-longest and most extensive mountain chain in Europe, stretching for ca. 1500 km across the central and eastern part of the continent. Being 100–350 km in width, it covers an area of ca. 190,000 km^2^ ^29^. This mountain chain extends mainly through Poland, Slovakia, Ukraine and Romania, but also encompasses small areas in Czechia, Austria, Hungary and, disputably, a fragment in Serbia across the Danube valley^30^. The Carpathians are known as a major biodiversity hotspot in Europe with a long history and strong tradition of natural history research^31^. Nevertheless, modern tools in ecology and evolution, especially those based on molecular markers, were introduced into the study of Carpathian biodiversity only during the last couple of decades, after the disappearance of the local geopolitical constraints^30^.

Located north of the Mediterranean Region, already in the Pleistocene permafrost zone and recurrently covered by glaciers in their higher parts, the Carpathians were not perceived, among the major European glacial refugia until recently. However, recently growing evidence that much of the area remained unglaciated during the Last Glacial Maximum (LGM), corroborated by results of palaeoecological and molecular phylogeographic studies, has largely altered such view (for an overview see^30,31^). Now it is generally accepted, that the Carpathians acted as refugium not only for temperate biota during the LGM but, during warmer episodes, also for the cold-adapted taxa, either at higher elevations (e.g.,^32,33^) or in habitats such as fens and peat bogs at lower elevations^34^. Most of the phylogeographic studies in the Carpathians focused on the terrestrial biota; however, the region has also been presumed as an important refugium for aquatic taxa. Already Malicky^35,36^, in his “dinodal theory”, predicted that cold-adapted and cold-tolerant freshwater biota would persist in suitable permanent habitats such as springs and headstreams, present in periglacial areas. Subsequent studies have proven this assumption for some aquatic insects, like the dipteran genus *Pedicia* Latreille, 1809^37^, riffle beetles^38^ or several caddisflies (e.g.,^31^).

Most recently, high lineage diversification in the Carpathian springs and streams, indicating long local divergence processes, even in the northernmost parts of the Carpathian Arc (i.e., the Western Carpathians), has been shown for the two gammarid morphospecies complexes widespread in Europe, i.e., *Gammarus balcanicus* ^20,24^ and *G. fossarum*^16,39^.

In this study we follow our former large-scale phylogenetic research^20^, which has revealed the Miocene phase of the Alpine orogenesis as the main factor influencing lineage divergence of *G. balcanicus* in the Carpathians. We aim at an in-depth exploration of the spatiotemporal divergence patterns and potential hidden diversity within this morphospecies in headwaters of the northernmost part of the Carpathian Arc, where it has its northern and northwesternmost range limit. In this region, *G. balcanicus* is the most common and abundant gammarid, particularly at higher altitudes^40^.

First, we hypothesise that various lineages survived Pleistocene glaciations in local periglacial refugia in the Carpathians from which, after LGM, some expanded to their present ranges. It should result in a mosaic pattern of lineage distribution with recently colonised areas showing low molecular diversity and the refugial regions with numerous divergent, locally endemic, lineages and high molecular diversity. Second, we hypothesise that some of these divergent lineages may represent cryptic or pseudo-cryptic species that may be defined and described via integrative methods.

## Results

Results of morphological and molecular analyses provided support for description and of a new species within the studied *G. balcanicus* species complex. The most unequivocal approach, was redescription of *Gammarus tatrensis* and description of new species—*G. stasiuki* sp. nov..

### MOTUs delimitation

**Figure 1:**
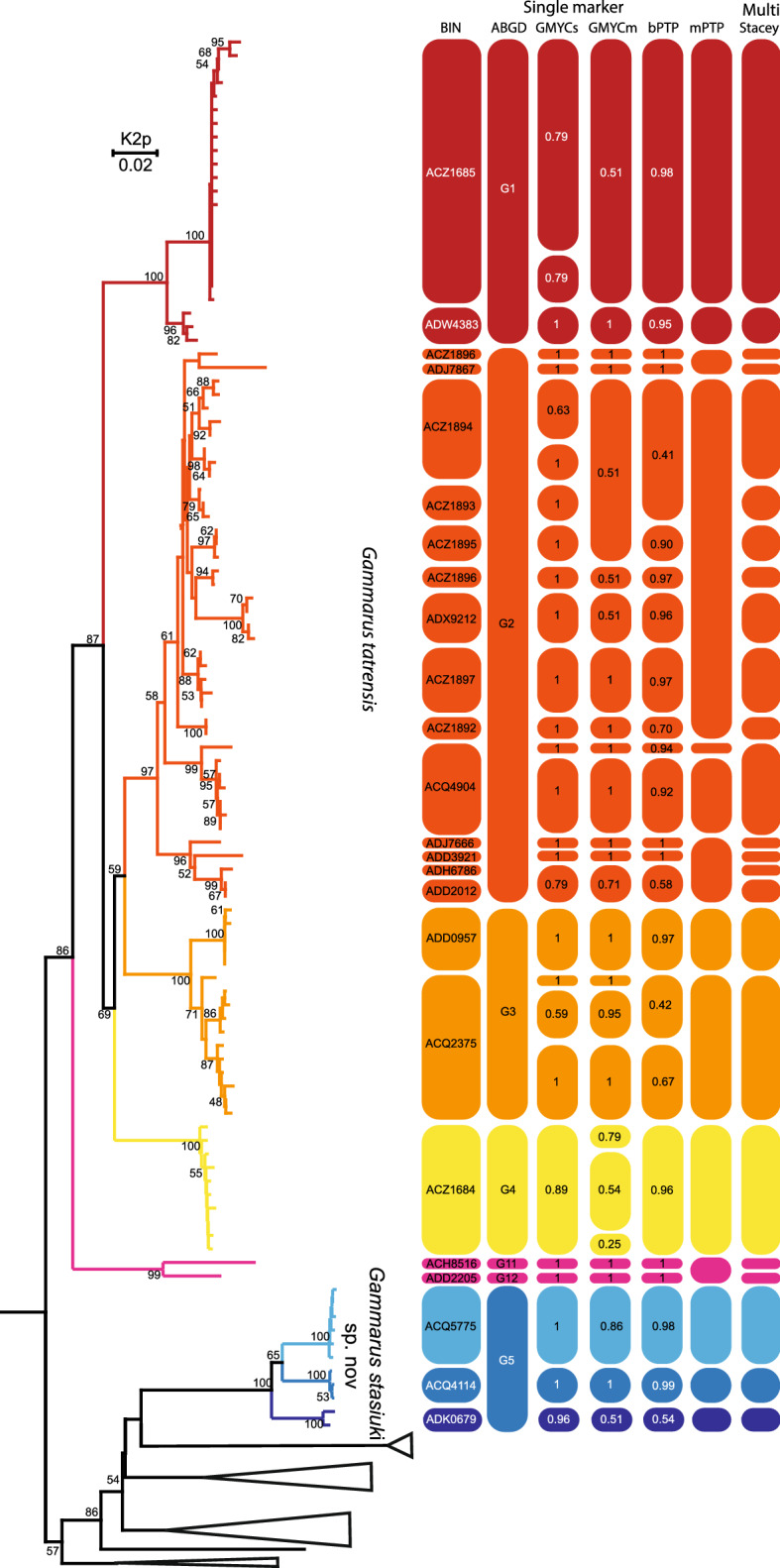
MOTUs delimitation. Neighbour-Joining tree constructed using COI haplotypes and Kimura 2-parameter distance. Bootstrap values > 50 annotated. Bars represent results of species delimitation methods. Outgroups collapsed.

Eight molecular operational taxonomical units (MOTU) delimitation methods provided partially similar results (Fig. 1). The BOLD clustering method revealed 21 BINs within *G. tatrensis* s.l. and 3 BINs within the *G. stasiuki* sp. nov. Current study provided also 12 additional BINs belonging to the sister lineage of the studied *Gammarus* species. The ABGD and ASAP provided similar results, among 10 resulting partitions *P* was stable for 0.0269–0.0437 (distribution of pairwise distances given in Fig. S2), with ASAP species threshold for preferred partition being: 0.0447 p-distance, the data was divided into 18 MOTUs (G1–G18). Six MOTUs fell within the *G. tatrensis* (G1–G4, G11, G12) and one within the *G. stasiuki* sp. nov. Sister lineages were classified in 12 MOTUs. There was no statistically significant difference between single and multiple GMYC methods (Chi^2^ = 4.654517, df = 6, *p* = 0.5888235). Both methods supported the hypothesis of multiple species (sGMYC: likelihood ratio = 44.497, *p* = 2 × 10^–10^, mGMYC: likelihood ratio = 49.152, *p* = 2 × 10^–11^). The sGMYC and mGMYC revealed 25 and 23 MOTUs respectively, for *G. tatrensis*, 3 for *G. stasiuki* sp. nov. and 14 for sister lineages. The PTP methods revealed different values for *G. tatrensis*: 21 (bPTP) vs. 11 (mPTP) as well as 14 bPTP vs. 9 mPTP in sister lineages. In the case of *G stasiuki* sp. nov., the number of MOTUs (3) was consistent for these two methods. The lowest posterior probability for MOTU grouping within bPTP was 0.43 while for mPTP it was 0.73. Multilocus species delimitation (STACEY) suggested the presence of 36 MOTUs (fraction 0.81) identical to BINs used as minimal clusters, from which 21 fell into *G. tatrensis* and 3 to *G. stasiuki* sp. nov. The same results were reported in additional analysis employing path sampling on multilocus data. The BIN delimitation had the highest marginal likelihood in comparison to delimitation using ABGD/ASAP or morphology (Table S2).

### Time calibrated reconstruction of phylogeny and distribution

**Figure 2:**
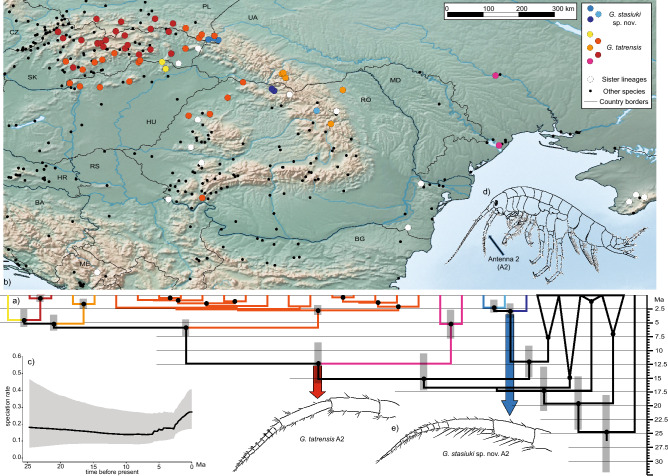
Phylogeny and distribution of *G. tatrensis*, *G. stasiuki* sp. nov and their sister lineages. (**a**) Time calibrated species phylogeny reconstruction based on the full multimarker data set generated in *BEAST. Species were defined using BINs (see Materials and Methods). White dots indicate nodes with a posterior probability (PP) > 0.75, grey bars on key nodes are showing a range of 95% HPD. Colours represent MOTUs. (**b**) Map of the Carpathians with sampling stations indicated by colour symbols. Colours represent MOTUs. White circles represent sister lineages of the studied species. Black dots represent sampling spots on which the studied group was not found (map constructed in QGIS 3—qgis.org, background obtained from: naturalearthdata.com). (**c**) Speciation rate through time inferred using BAMM tools. (**d**) General body shape of gammarid. (**e**) Antenna 2 of *G. tatrensis* and *G. stasiuki* sp. nov.

The saturation test revealed no significant loss of phylogenetic signal for any of the tested markers (ISS < ISS.cSym, *p* < 0.00). The Neighbor-Joining distance tree reconstructed for each marker separately resulted in weakly resolved topologies for nuclear markers with few well-supported clades and strong phylogenetic signal for mitochondrial markers, especially COI. The analysed markers did not convey conflicting phylogenetic signals (Fig. S1). The multi-marker reconstruction of phylogeny (Fig. 2a) showed that both *G. tatrensis* and *G. stasiuki* sp. nov. form well supported monophyletic clades. Both species separated from other lineages of *G. balcanicus* species complex already in the Middle Miocene. Soon after, the lineage of *G. tatrensis,* endemic to Ukrainian Lowlands, diverged from the others. The remaining four lineages started to diverge in Late Miocene/Early Pliocene, while the intra-lineage divergence is dated to Late Pliocene/Pleistocene. *G. tatrensis* is now the most widely distributed species in the northern Carpathians (Fig. 2b). Its distribution reaches Western/Southern Carpathians, Apuşeni Mts. and Transylvanian Plateau. The divergence of *G. stasiuki* sp. nov. into three lineages is dated to Late Miocene/Early Pliocene. Its distribution is limited to several locations in the Eastern Carpathians. Except for a few locations in Poland the species was found in northern Romania.

### Speciation rate changes through time

Bayes factors showed that the model with one rate shift is preferred (BF > 6) over the null model with 0 shifts. The shift of diversification occurred at the divergence of *G. tatrensis* Carpathian lineages in the Late Miocene (Fig. 2c, Fig. S3). The BAMM plot illustrating changes in diversification rates shows an increase in the Late Miocene and the following fluctuation ended at the beginning of the Pliocene with strong increment (Fig. 2c). These increments are also visible on the LTT plot (Fig. S3), however, the pick of diversification there is not so prominent.

### Demographic analysis

**Figure 3:**
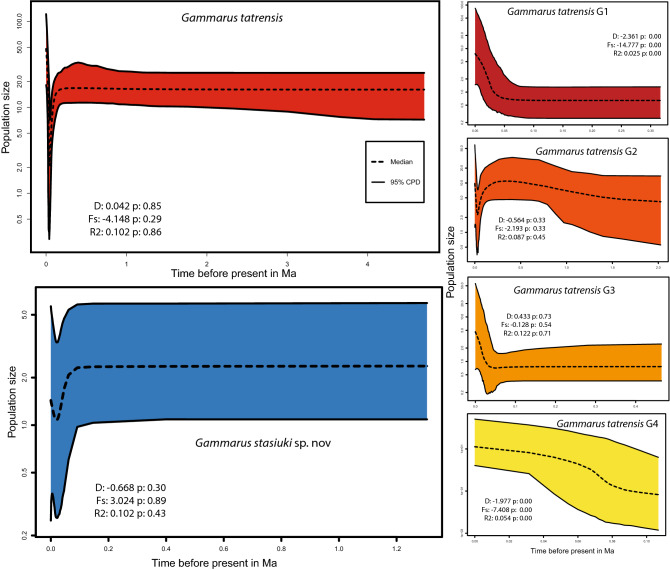
Demographic history of *G. tatrensis* and *G. stasiuki* sp. nov. represented through extended Bayesian skyline plots (eBSP) based on COI. Neutrality tests presented on figure: Tajima’s D, Fu’s Fs and Ramos-Onsins and Rozas's R2.

All MOTUs of *G. tatrensis* revealed by ABGD show some signs of postglacial demographic expansion (Fig. 3). This is particularly evident for MOTU G1 where population growth after putative bottleneck event is supported statistically. MOTU G4 shows only mild population size increment but the significance of neutrality tests suggests also an impact of bottleneck effect on its molecular diversity. Population size growth after decline shown on eBSP of MOTU G2 is not supported by neutrality tests. MOTU G2 is showing a possible bottleneck during the most recent glacial maximum. Lack of statistical support for demographic growth is also observed for MOTU G3. *Gammarus stasiuki* went through a bottleneck during the Last Glacial Maximum, and the population size has not fully recovered, but these changes are not significant.

### Network reconstructions

**Figure 4:**
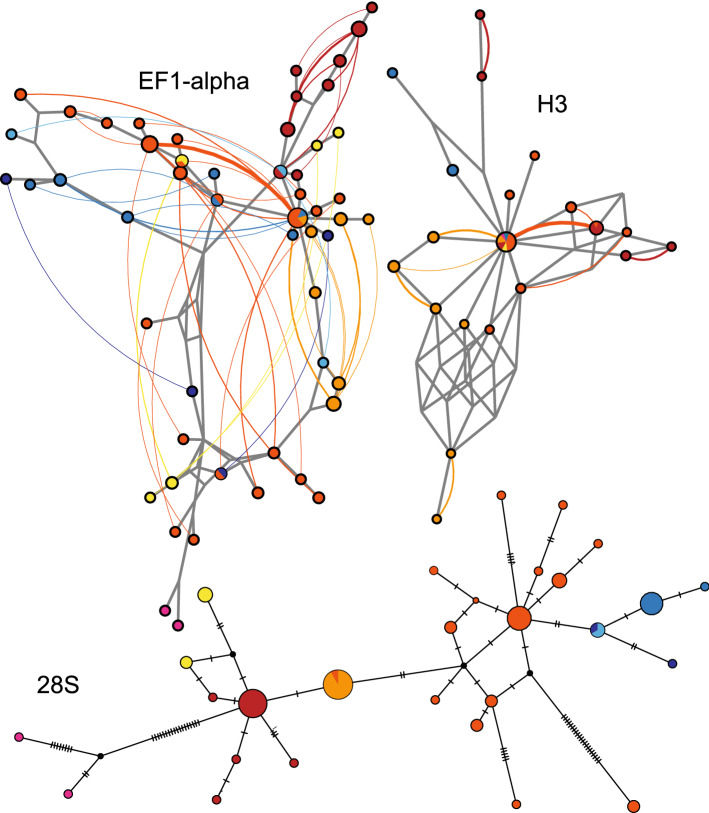
Haplotype networks for nuclear markers based on all *G. tatrensis* and *G. stasiuki* sp. nov. sequences. Networks for EF1-alpha and H3 reconstructed with phased haplotypes in haploweb software. 28S haplotype network reconstructed using the median-joining approach. Colours represent MOTUs (see Figs. 1, 2).

The haploweb of EF1-alpha revealed the existence of a complex pattern among the studied lineages (Fig. 4). All lineages belonging to *G. tatrensis*, with exception of those from the Ukrainian Lowlands (G11, G12), share haplotypes. Only partial distinctiveness can be observed for the lineage G1. The haplotypes of *G. stasiuki* sp. nov. are, in general, intermingled with *G. tatrensis*, with partial distinctiveness of haplotypes belonging to BIN ADK0679. A similar pattern is observed in haploweb reconstructed for the H3 nuclear marker, in which almost all MOTUs share haplotypes, including those from the Ukrainian Lowlands. The network of 28S rDNA shows a separation of *G. tatrensis* from *G. stasiuki,* the distinctiveness of most of the *G. tatrensis* MOTUs, and clear divergence of the lineage inhabiting the Ukrainian Lowland from the others within *G. tatrensis*.

### Species description

***Gammarus stasiuki ***sp. nov Jażdżewski K., Mamos T., Grabowski M.

*Gammarus balcanicus* form B^41^, pp. 51, 90–91; fig 6 L, M, fig 8 D, E, F, I, K, Fig. 23. Locality—Bieszczady Mts. *Gammarus balcanicus* form B^42^, p. 76; fig. 1 D, E, F, fig. 3. Locality—Bieszczady Mts. *G. balcanicus* form B^43^, pp. 61, 64, 69; fig. 1. Locality—Dwernik = Prowcza stream, Bieszczady Mts., 1020 m a.s.l. *G. balcanicus* form B;^44^, p. 131, fig. 1, p. 137, fig. 6. Locality—four streams in south-easternmost Poland. *Gammarus balcanicus* form B^45^, pp. 36–39; fig. 2. Eleven localities in Bieszczady National Park.

### Etymology

We name this species in honour of Andrzej Stasiuk, a very successful and internationally acclaimed Polish writer, journalist and literary critic. By this we pay tribute to his travel literature and essays that describe the natural and cultural environment of Eastern Europe, including the Carpathians, where he has chosen to settle.

### Material examined

The morphologically examined material consists of samples coming form 4 locations spread along the Eastern Carpathians (Poland and Romania, details on locations in BOLD dataset: DS-GAMNCARP). The examined samples consist from over 200 specimens and are stored in the Department of Invertebrate Zoology and Hydrobiology, University of Lodz (KZBiH, UniLodz).

TYPE MATERIAL and LOCUS TYPICUS: , the t ype sample was collected in the locality Mała Rawka Mt. (N 49.1135, E 22.5763), 1140 m a.s.l., Poland, on 23.04.2016, by M. Grabowski and T. Mamos. The Holotype, (male, 13 mm) is stored in Museum and Institute of Zoology Polish Academy of Sciences (MIIZ PAN) under accession code: MIZ PAN CRU 1. Additional non-type material stored in MIIZ PAN consist of over 20 specimens (males and females), microscope slides and DNA isolate (MIZ PAN CRU 2- MIZ PAN CRU 26). The new taxon name and status are registered in the Official Register of Zoological Nomenclature (ZooBank). ZooBank Life Science Identifier (LSID): urn:lsid:zoobank.org:act:1AFD0835-BE27-42FF-9B68-59D37CBEC237.

### Description

**Figure 5:**
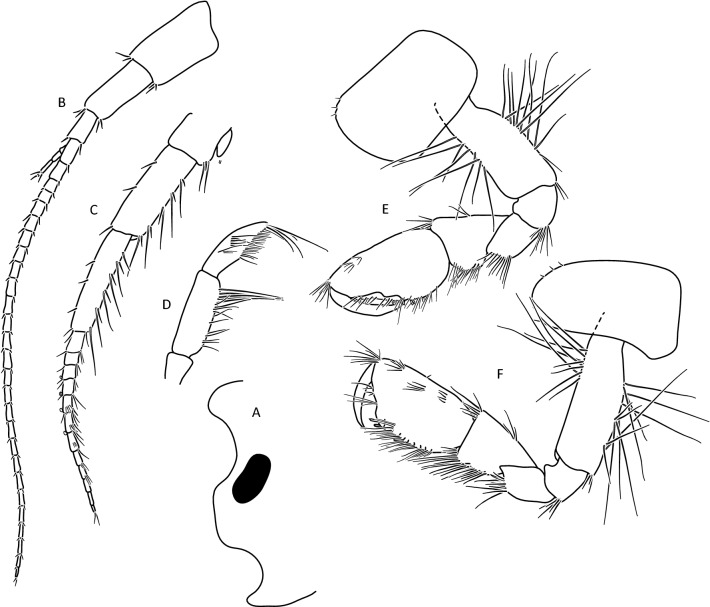
*Gammarus stasiuki* sp. nov., male—12 mm. (**a**) left side of head, (**b**) antenna 1, (**c**) antenna 2, (**d**) mandibular palp, (**e**) gnathopod 1, (**f**) gnathopod 2.

**Figure 6:**
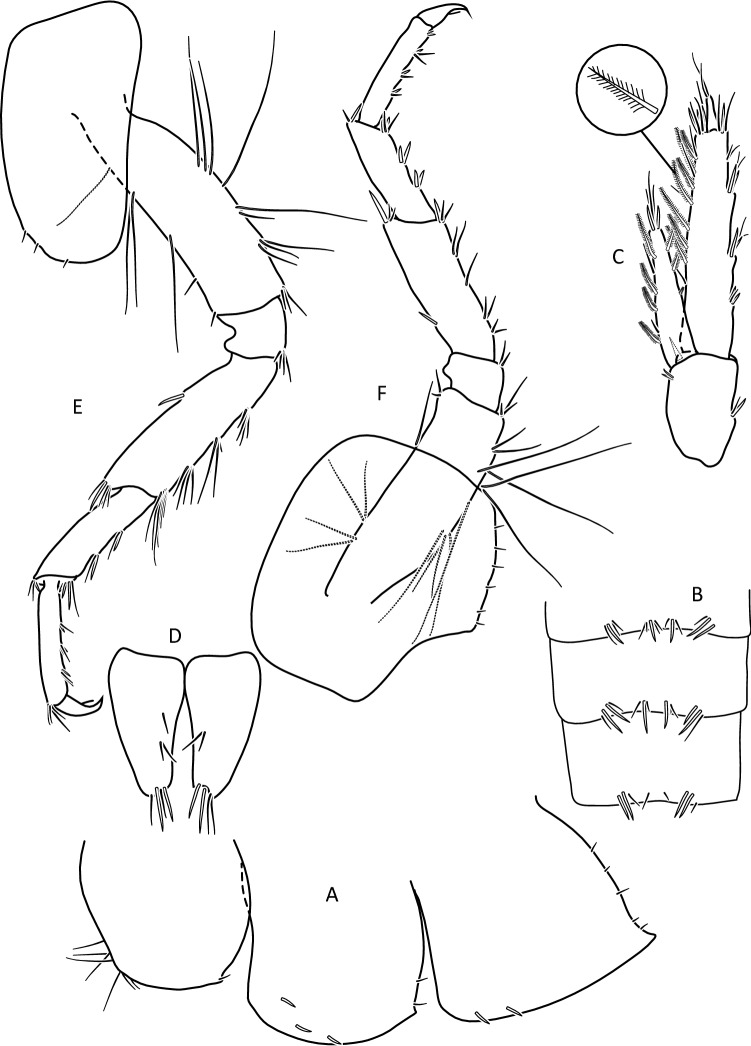
*Gammarus stasiuki* sp. nov., male—12 mm. (**a**) epimera I-III, (**b**) urosome, (**c**) uropod 3, (**d**) telson, (**e**) pereopod 3, (**f**) pereopod 4.

**Figure 7:**
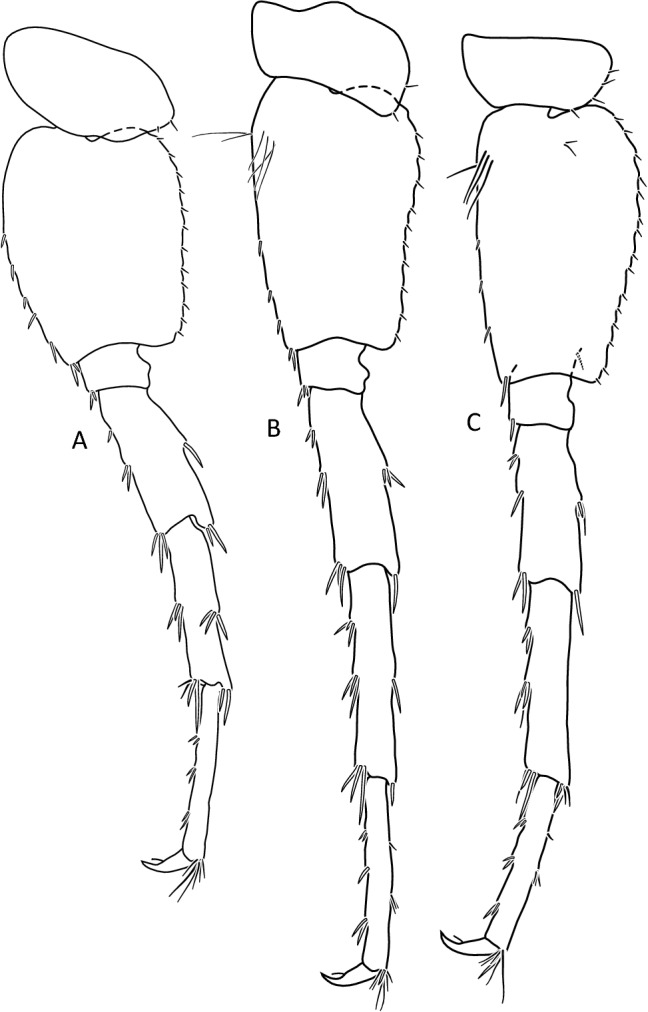
*Gammarus stasiuki* sp. nov., male—12 mm. (**a**) pereopod 5, (**b**) pereopod 6, (**c**) pereopod 7.

**Figure 8:**
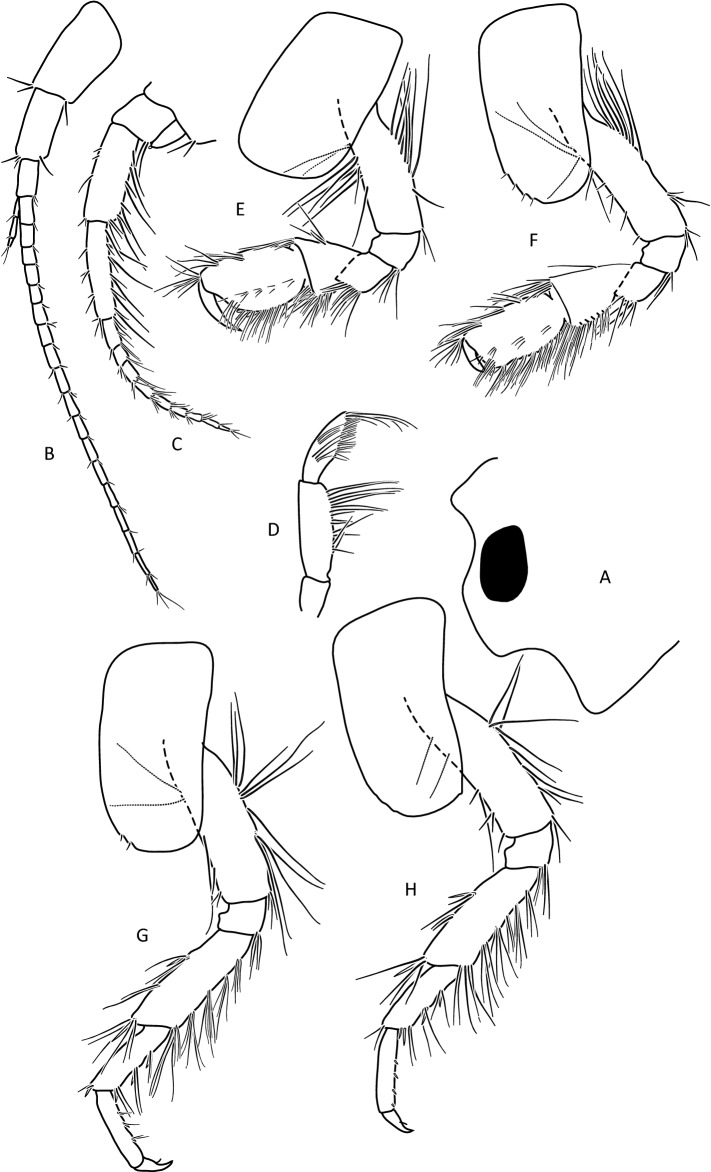
*Gammarus stasiuki* sp. nov., female—10 mm. (**a**) left side of head, (**b**) antenna 1, (**c**) antenna 2, (**d**) mandibular palp, (**e**) gnathopod 1, (**f**) gnathopod 2, (**g**) pereopod 3, (**h**) pereopod 4.

The morphological analysis supported discrimination of *G. stasiuki* sp. nov. and *G. tatrensis* based on setation of the second antenna (Figs. 1, 2e). The examination of ultrastructure through SEM did not show any features delimiting the taxa or MOTUs (Fig. S4). Male (Figs. 5, 6, 7, 8): max. length observed 13 mm. The length of A 1 length equal to the joint length of head and first four pereon segments, A 2 somewhat shorter—head + 3 pereon segments. Head lateral lobe rounded, eyes medium size, oval or reniform; eye length sub-equal to the A 1 basal width (Fig. 5a,b). A 1 main flagellum with 20–25 articles and accessory flagellum with 3–4 articles (Fig. 5b). A 2 flagellum with 10–12 articles; in adult males (over 10 mm) with 4–5 calceoli on 4 to 8 flagellum articles, often poorly visible (Fig. 5c). Third article of mandibular palp with brush of over 20 D-setae forming even row (Fig. 5).

**Figure 9:**
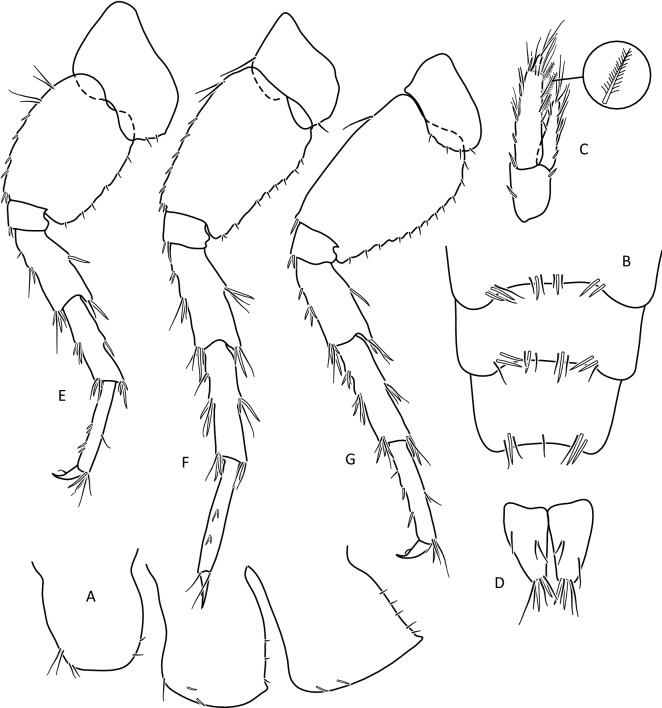
*Gammarus stasiuki* sp. nov, female—12 mm. (**a**) epimera I-III, (**b**) urosome, (**c**) uropod 3, (**d**) telson, (**e**) pereopod 5, (**f**) pereopod 6, (**g**) pereopod 7.

Lower margins of basal A 2 articles 4 and 5 richly setose, with 5–6 groups of 2–4 setae. Length of longest setae sub-equal to the width of 4th article and somewhat longer than the width of 5th article (Fig. 5b). Posterior margins of gnathopod’s carpus and propus richly setose (Fig. 5d,e). P 3 merus posterior margin with 4–5 groups of numerous setae as long or a bit longer than merus width (Fig. 5a). Basis of P 6 and P 7 with posterior margin crenulated, in crenules a small setule. Distal part of basis of these pereopods 1.5 times wider than ischium width. Distoposterior lobe of basis P 6 and P 7 sub-rectangular. Posterior margin of P 6 basis somewhat concave, posterior margin of P 7 basis slightly convex (Fig. 5e,f). Second epimeral plate 2 (E2) rather characteristic, with lower margin distinctly convex ending with a small tooth; posterior margin of E2 slightly to distinctly convex (Fig. 6a). Urosomites 1–3 with two medial and two lateral groups of 1–3 spines and/or 1–2 setules (Fig. 6b). Uropod 3 biramous, endopodite length ca. 2/3 of exopodite length (Fig. 6c). Outer margin of the first article of U 3 exopodite with 3 groups of 1–2 spines and 1–4 setae, some a bit longer than spines. Apically this first article of exopodite with 3–5 spines and several setae, some are longer than spines. Second exopodite article apically with 3 setae, the longest as long as this article. Endopodite of U 3 apically with 1–2 spines and 3–4 setae, some over 2 times longer than spines. Inner margin of U 3 exopodite with several groups of 1–3 setae, ca. half of these setae are feathered. Outer margin of U 3 endopodite with 3–5 groups of spines and setae, of which 3–4 are feathered. Inner margin of U 3 endopodite with 2–3 groups of setae, several setae are feathered (Fig. 6c). Feathered setae are sometimes broken. Telson lobes with apical 2–3 spines and 3–4 setae, some a bit longer than spines. On the surface of telson lobe 1–4 subapical and/or subbasal setae (Fig. 6d), very rarely 1 spine. Female (Figs. 8, 9): max. length observed 11 mm. Clear sexual dimorphism in the setation of appendages: setae on the lower margin of A 2 peduncular articles 4 and 5 distinctly longer than in males; in 4th article setae are 1. 5 × longer than this article width and in 5th article two times longer than this article width (Fig. 8c). Similarly, in females, P 3 merus is more setose, with setae twice as long as the merus width (Fig. 8g). In the U 3 exopodite outer margin longest setae are 2 times longer than spines. Also, apical setae of U 3 endopodite can be 3 times longer than accompanying spines (Fig. 9c). The body surface, plate margins and appendages in juvenile specimens are less dressed with spines and setae than in adults.

DISTRIBUTION and ECOLOGY: The species is found only in a few locations in Eastern Carpathians: Bieszczady Mts. (Poland), Maramureș Mts.(Romania) and Călimani Mts. (Romania). It can be found in small cold mountain streams with rock bottom and leaf litter.

### Gammarus tatrensis (S. Karaman, 1931)—redescription

*Rivulogammarus tatrensis*; S. Karaman 1931^3^, pp. 97–98, figs 4 a, b; localities: (1) Ďumbier Mt. (Low Tatra Mts, Slovakia); (2) Kuzy, Carpathian Mts, western Ukraine (probably not *G. tatrensis*).

*Gammarus (Rivulogammarus) balcanicus tatrensis* (S. Karaman) 1931^3^ (sic!)^46^, pp. 218–222, figs 4 A-D, locality—Tatra Mts, several streams in eastern Slovakia.

*Gammarus (Rivulogammarus) balcanicus* subsp. *tatrensis*^47^, p. 256, several streams in western Ukraine, Carpathian Mts (probably not *G. tatrensis*).

*Gammarus (Rivulogammarus) balcanicus tatrensis* S. Karaman 1931 (sic!)^48^, pp. 562–567, Tab. LXXVI fig 3, Tab.LXXVII figs 3–4.

*Gammarus balcanicus tatrensis* (S. Karaman, 1931)^49^, fig 2; numerous localities in northern Carpathian Mts in Slovakia, i.a. Vyšná Boca near Ďumbier Mt, collected at 7.07.1954 year.

*G. b. tatrensis* (S. Kar.) and *G. balcanicus* form A^41^, fig 8a,b; numerous localities in Polish Carpathian Mts and sub-Carpathian region.

### Material examined

The morphologically examined material consists of 54 samples each representing a single location collected all along the Carpathian Arch and Ukrainian Lowlands (details: DS-GAMNCARP). The examined samples were collected in 2009, by D. Zielinski and in 2008, 2016 by T. Mamos and M. Grabowski. The examined material consists of over 2000 specimens and is stored in KZBiH, UniLodz.

TYPE MATERIAL and LOCUS TYPICUS: the type sample was collected in the locality Vyš ná Boca at the Ďumbier Mt, 1030 m a.s.l. (N 48.923, E 19.736), Slovakia, on 15.05.2015, by all the authors . The Holotype, (male, 13 mm) is stored in MIIZ PAN under accession code: MIZ PAN CRU 27. Additional, stored in MIIZ PAN, non-type material consists of over 3 specimens (males and females), microscope slides and DNA isolate (MIZ PAN CRU 28—MIZ PAN CRU 57). The new taxon name and status are registered in ZooBank: urn:lsid:zoobank.org:act:B5513CA2-B93F-49AC-9F3E-4E84C8F59349.

### Redescription

**Figure 10:**
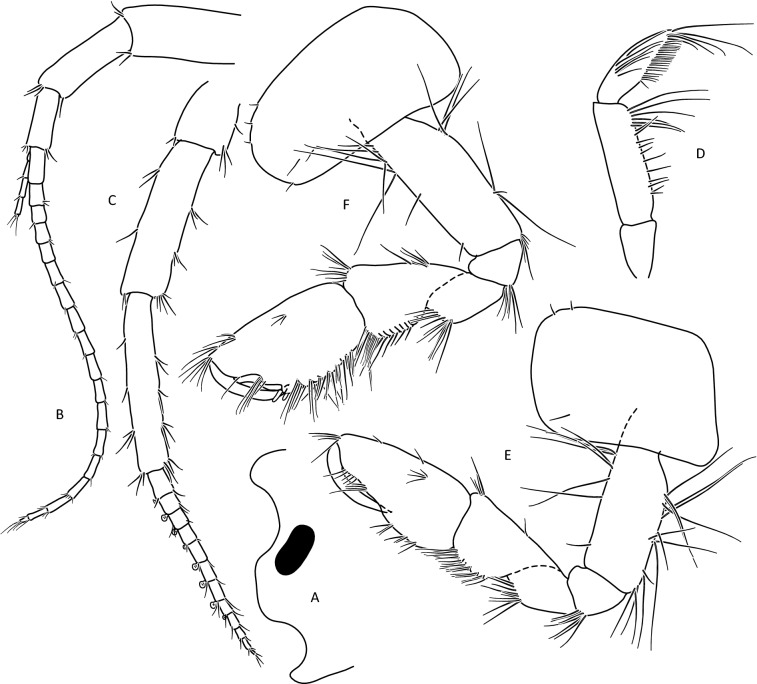
*Gammarus tatrensis*, male—14 mm. (**a**) left side of head, (**b**) antenna 1, (**c**) antenna 2, (**d**) mandibular palp, (**e**) gnathopod 1, (**f**) gnathopod 2.

**Figure 11:**
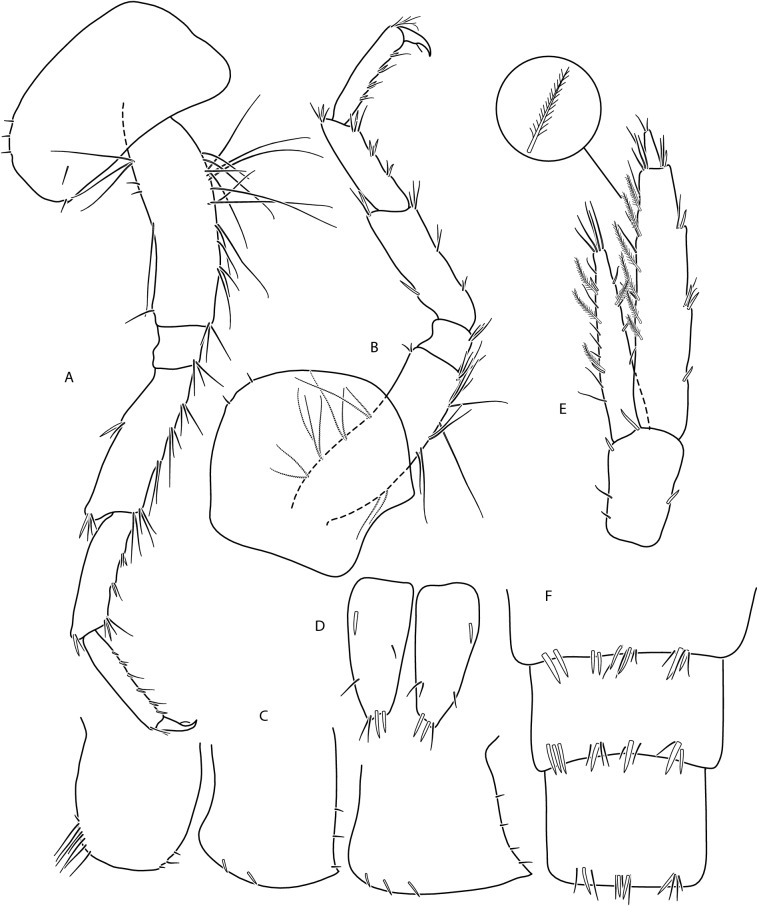
*Gammarus tatrensis*, male—14 mm. (**a**) pereopod 3, (**b**) pereopod 4, (**c**) epimera 1- 3, (**d**) telson, (**e**) uropod 3, (**f**) *Gammarus tatrensis*, male—12 mm urosome.

**Figure 12:**
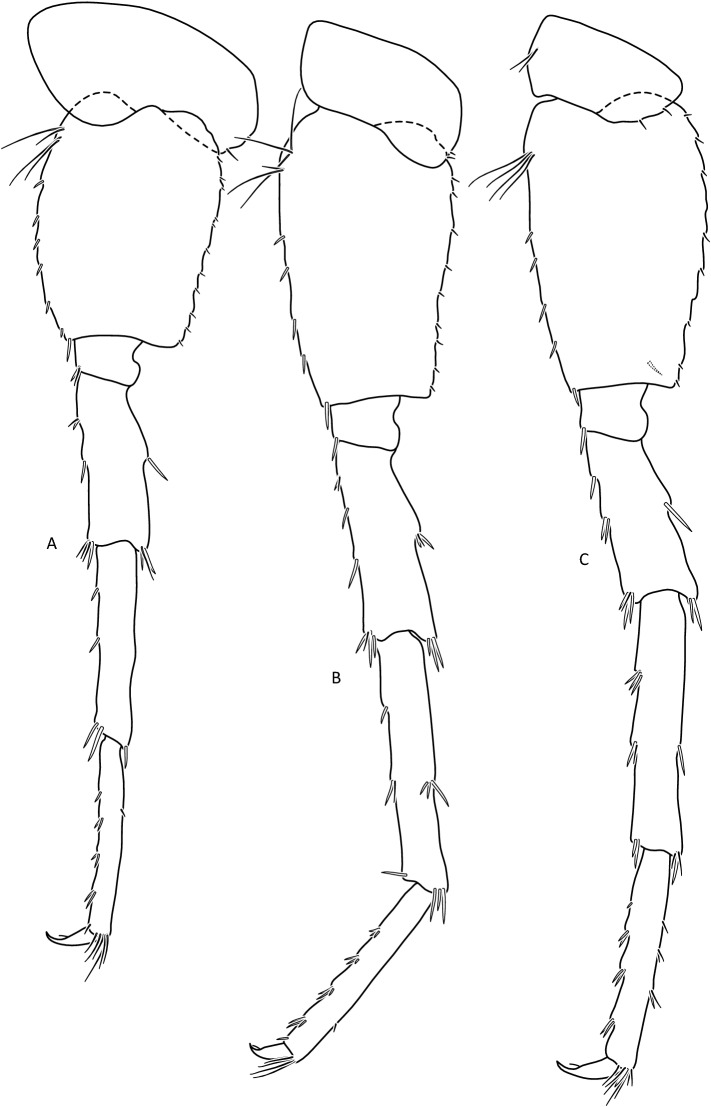
*Gammarus tatrensis*, male—14 mm. (**a**) pereopod 5, (**b**) pereopod 6, (**c**) pereopod 7. Female (Figs. 13, 14, 15): max. length observed 13 mm. Setae on the lower margin of A 2 peduncle articles 4 and 5 longer than in males; the longest setae of the 4th article are a bit longer than this article width; longest setae of 5th article are up to 1. 5 × times longer than this article width (Fig. 13b). Posterior margin of pereopod 3 merus richly setose—in 3–4 groups altogether 10–15 setae (Fig. 14a); the longest 1. 5 × longer than merus width.

**Figure 13:**
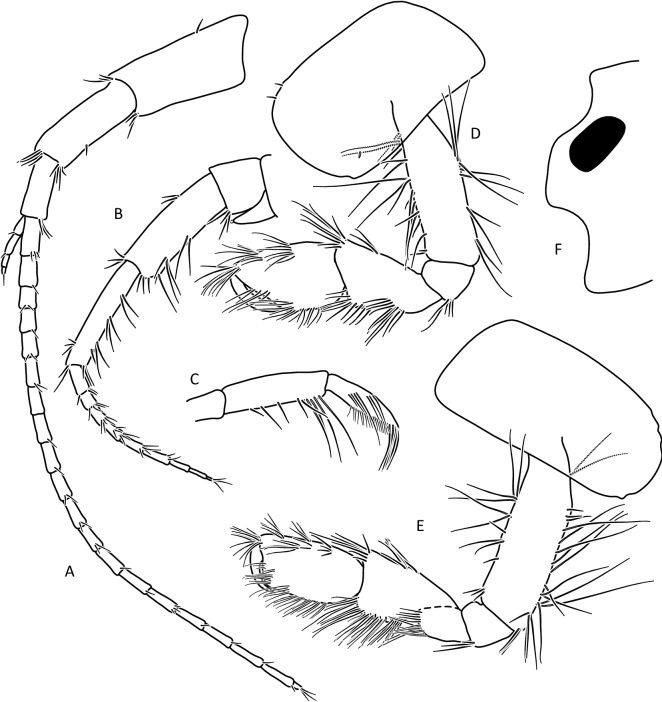
*Gammarus tatrensis*, female—12 mm. (**a**) antenna 1, (**b**) antenna 2, (**c**) mandibular palp, (**d**) gnathopod 1, (**e**) gnathopod 2, (**f**) head.

**Figure 14:**
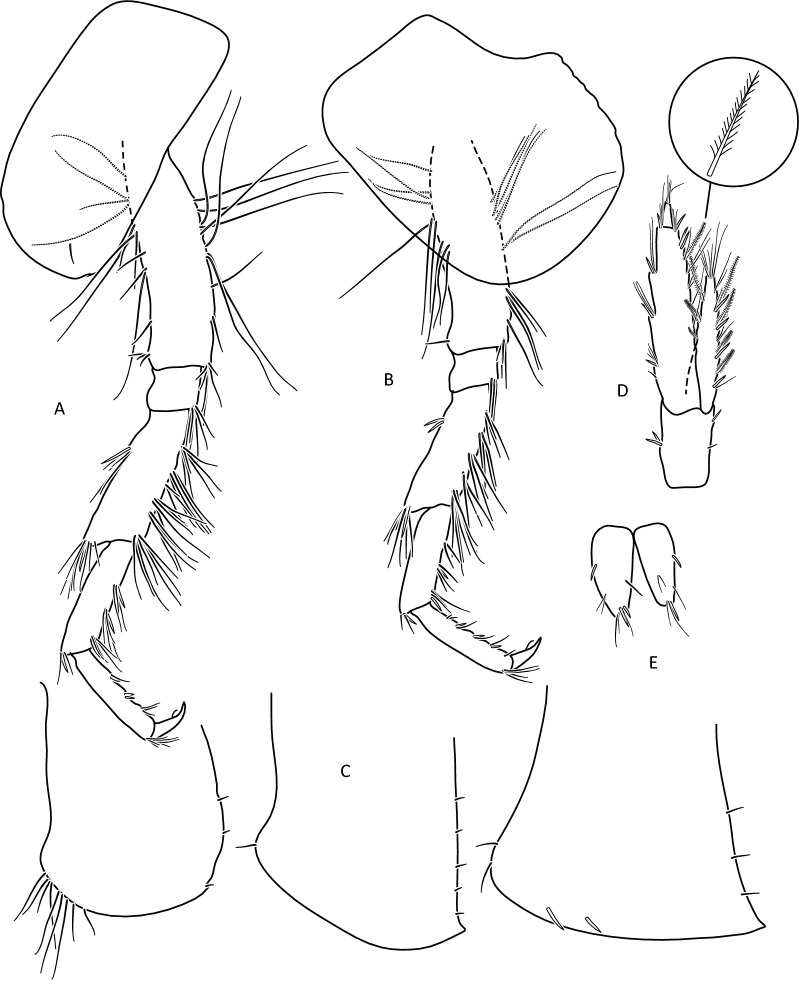
*Gammarus tatrensis*, female—12 mm. (**a**) epimera I-III, (**b**) urosome, (**c**) uropod 3, (**d**) telson, (**e**) pereopod 5, (**f**) pereopod 6, (**g**) pereopod 7.

**Figure 15:**
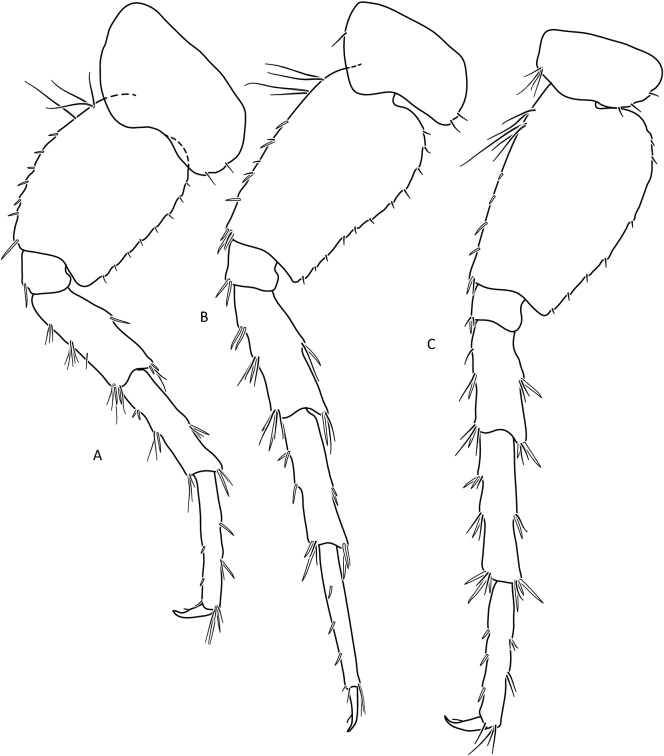
*Gammarus tatrensis*, female—12 mm. (**a**) pereopod 5, (**b**) pereopod 6, (**c**) pereopod 7.

Male (Figs. 10, 11, 12): max. length observed 14 mm. Head lateral lobe rounded, eyes oval or reniform (Fig. 10a); eye length equal to the A I basal width. Length of A I greater than the length of head and first four pereon segments, A 2 of the length of head and over 3 pereon segments. A I flagellum with articles 20–30, accessory flagellum with 3–4 articles. Flagellum of A 2 with 10–12 articles. Calceoli set on basal articles are present only in males larger than 9 mm. Mandibular palp with brush of over 20 D-setae in even row. Lower margin of A 2 basal articles (4 and 5) poorly setose: 4th article with 2–3 groups of short setae (ca. 0.5 of the article width) and 5th article with 3–4 groups of setae, their length equal to the article width (Fig. 10c). Posterior margin of merus of pereopod 3 with 3–4 groups of setae, the longest is equal to merus width (Fig. 11a). Posterior margin of basis of peropod 6 and 7 crenulated with small setules; this margin in P 6 slightly concave, in P 7 slightly convex. Distal part of these basis articles 1. 5 times wider than ischium width; their distoposterior lobe rectangular (Fig. 12b,c). Lower margin of epimeral plate 2 slightly convex, posterior margin straight or slightly concave; posterodistal tooth of E 2 medium size. Epimeral plate 3 with strongly concave posterior margin and its posterodistal part is distinctly produced (Fig. 11c). Each urosomite segment dorsally with 4 groups of spines and/or setules; two central groups in urosomite 3 are near each other. Lateral groups usually with 2 spines and 1–3 setules, central groups usually with 1 spine and/or 2–3 setules (Fig. 11e). Uropod 3 biramous, endopodite length *ca* 2/3 of exopodite length. Outer margin of the first exopodite article usually with 3 groups of spines accompanied by 1–2 short setae, sometimes longer than spines; rarely on this exopodite margin sub-apically there is fourth group of only setae. Exopodite first article apically with 2–3 spines and several short setae; exopodite second article apically with 2–4 short setae. Inner margin of U 3 exopodite with several groups of setae, several of these setae are feathered. Endopodite apically with 1–3 spines and 3–4 setae; both inner and outer margin of endopodite with several groups of setae, sometimes accompanied with a spine; several setae are feathered (Fig. 11f). Telson lobes apically with 1–3 spines and 2–3 setules. On telson lobes one subbasal and sometimes one subapical spine and/or 1–2 setules (Fig. 11d). Female (Figs. 13, 14, 15): max. length observed 13 mm. Setae on the lower margin of A 2 peduncle articles 4 and 5 longer than in males; the longest setae of the 4th article are bit longer than this article width; longest setae of 5th article are up to 1. 5 × times longer than this article width (Fig. 13b). Posterior margin of pereopod 3 merus richly setose—in 3–4 groups altogether 10–15 setae (Fig. 14a); the longest 1. 5 × longer than merus width.

DISTRIBUTION and ECOLOGY: The species has wide distribution in Western Carpathians but also can be found in some locations on Eastern, Southern Carpathians and Ukrainian Lowlands. Species can be found on different altitudes but predominantly in mountain streams with rock bottom and abundant leaf litter substrate.

### Remarks on the original description

S. Karaman^3^ has described *Rivulogammarus tatrensis* in ver y brief form, including only a limited number of features. Therefore we have decided to make a full morphological redescription of the species. T he samples used in the original description were collected at the Ďumbier Mt. (Low Tatra Mts, Slovakia, Czechoslovakia at that time) and in the locality Kuziy Massif (western-most Ukraine—Eastern Carpathians). In redescription we are using material from the first location noted as locus typicus in the original description. However we did not have material from the second location (Ukraine) mentioned by S. Karaman, according to our examination of numerous samples collected along the Carpathian Mts, it is highly possible that another MOTU of *Gammarus tatrensis* may occur there.

## Discussion

The Carpathian Arch is recognised as one of the most important extra-Mediterranean European glacial refugia^50^. However, so far the majority of studies suggested that it concerned mostly the Southern and Eastern Carpathians (for a summary see^31^). Moreover, some studies rejected the existence of northern “cryptic” glacial refugia^51^. In the current study, we provide clear evidence that two species, belonging to the *G. balcanicus* species complex, survived through the Ice Age in northern refugia within the Carpathians. Moreover, within the most widely distributed species, *G. tatrensis*, several distinct MOTUs could be distinguished, which survived the Ice Age in separate refugia. The MOTUs, whose evolutionary history reaches the Miocene, are mostly endemic to the northern area of the Carpathians. Only MOTU G2 is widely distributed around the Danube drainage and is characterised by high genetic diversity, the presence of locally endemic haplogroups and private haplotypes. The range of this MOTU extends through the periglacial region.

The most striking case of survivalist is MOTU G1, whose present distribution lies within the area covered, in the highest parts, by a glacier during the Last Glacial Maximum. Such a survival of old lineages in the northern Carpathian refugia was also reported for *G. fossarum* morphospecies^39^, as well as for *G. leopoliensis*, one of the lineages from within the *G. balcanicus* complex^20,23^. The existence of glacial refugia in the northern Carpathians is evidenced by Pleistocene fossils of land forest-dwelling molluscs (e.g., *Orcula dollium*^52^), which supports the hypothesis of broadleaf forest micro refugia in the region^53^. Organic debris, such as leaves, is known as the primary diet component for amphipods of the genus *Gammarus*^54^ and could provide a food source even through the Last Glacial Maximum. Moreover, compared to other European species, the *G. balcanicus* complex consists of amphipods that can be usually found in higher altitudes, especially in the northern regions of the Carpathians^21,24,40^, therefore being adapted to well-oxygenated and low-temperature habitats. Thanks to such adaptation, survival in cold, northern refugia may have been more likely for the species complex.

The increase of diversification rate of *G. balcanicus* complex in the northern Carpathians through Pleistocene suggests that Ice Age could be a driving force of local lineages speciation. Such proliferation of their diversity could be possible due to climate oscillation and survival of different lineages in multiple cryptic refuges. Such survival, during adverse conditions, could be possible in the periglacial zone. In fact, large areas of the northern range of the Carpathians consisted of periglacial areas, not covered permanently by ice sheet. It could thus provide suitable habitats for *Gammarus* sp. survival^55^ (and references therein). It also seems that glaciations were the main factor behind demographic changes of the local lineages. These changes can be deduced in both studied species and their MOTUs. For example, in the Western Carpathians, MOTU G1 of *G. tatrensis* and *G. stasiuki* sp. nov. experienced a putative population bottlenecks at the end of the Pleistocene (*G. stasiuki* sp. nov. only according to eBSP). These lineages are distributed at northern locations and, therefore, probably more affected by the Pleistocene glaciations. According to neutrality tests, also MOTU G4 could putatively go through the bottleneck effect. However, this is not supported by the eBSP plot. On the contrary, MOTU G3, distributed only in the Eastern Carpathians, does not have any signs of the negative impact of glaciation on its population size. In general, we deduce a relatively stable population size through the Pleistocene with a post-Pleistocene increase of the population size (particularly MOTUs G1, G2 and G3) that could be related to postglacial dispersal and colonisation processes. The relatively constant population size, revealed for the lineage G4, is probably related to the fact that it is present in low altitudes and beyond the glacial reach in the Pleistocene. The absence of a decrease in population size and postglacial increment was already observed in the case of other gammarids: *G. fossarum*^39^ and *G. jazdzewskii*^16^. The proliferation of populations in periglacial regions was also reported in the case of *Asellus aquaticus* all over Europe^56^. In contrast to *G. tatrensis, G. stasiuki* has not gone through a noticeable demographic expansion after the Pleistocene and has remained limited geographically. We cannot discard that this pattern is a result of a competitive displacement by *G. tatrensis* in their common ecological niche.

Definition of species borders has been a topic of discussion for decades and still is controversial. In our study, we decide not to tackle the different concepts but to base species hypothesis on the integrative morphological and molecular approach. Results of the study provides evidence for a species new to science and for resurrecting another one (see above for taxonomic discussion) within the *Gammarus balcanicus* complex in the northern Carpathians. However, our results show that there are more MOTUs that could represent putative cryptic or pseudo-cryptic species. Hopefully, in the future, new methods based on anatomical features, ecology, ethology or secreted pheromones will help in delimitation, identification and definition of such putative species. So far, our study reveals that molecular analysis, primarily based on COI barcoding, is a fast and handy, even if only provisional, method of initial species delimitation within such “taxonomically difficult” animal groups. It should be used as a first step of the species delimitation process to propose primary species hypotheses (see also^28^). Our study is improving an online reference library of DNA barcodes accessible through BOLD^57^. Provision of well-curated data sets (available within reference libraries), such as the one from the current study, fills gaps in the knowledge of cryptic biota. Such activities are of utter importance in founding the base for future biodiversity assessments (for an overview see^58^).

In recent years, substantial cryptic diversity in numerous taxa, especially among gammarid crustaceans, was discovered using molecular markers (e.g.,^22,25,59^). The urgency for the classification of newly recognised cryptic species remains largely unchallenged while being essential for planning rational and effective conservation strategies^60,61^. On the other hand, we lack a universal and widely acceptable definition of cryptic species, followed by proper delimitation and diagnostic methodology.

The results of our study show that integrative approach, can reveal important patterns in diversity. We demonstrate that the two species, *G. tatrensis* and *G. stasiuki* sp. nov., are extremely similar to each other in terms of morphology and there is only one reliable feature that allows to distinguish them. Unfortunately, using SEM to detect possible differences in cuticle ultrastructure does not provide any additional information. Instead, the molecular species delimitation methods show a plethora of MOTUs that may, hypothetically, represent separate species. However, while the mtDNA shows categorical and geographically well-structured patterns of diversification, nuclear markers show a less clear picture. Despite that, in all nuclear markers the divergence between haplotypes is noticeable, in the case of EF1-alpha and H3 these haplotypes are shared between mitochondrial MOTUs. In contrast, the third nuclear marker, 28S rDNA, corroborates the delimitation of species and MOTUs.

Such discrepancies between gene histories and putative species borders are commonly attributed to gene flow between species, i.e., hybridization and to incomplete lineage sorting—retention of ancient polymorphism^62,63^. Retention of ancestral polymorphisms between species is especially well documented in the case of an adaptive radiation process (review in^64^). In the light of our findings, it seems that the mechanism may be more frequently found in various taxa^63^. However, proper identification of the drivers of speciation in the studied group will require the use of wide genome sequencing of multiple lineages.

On one hand, sharing of haplotypes is common feature of slowly evolving markers, when related taxa are considered. On the other hand, COI cannot be said to be a universal solution either. In many taxa (e.g., Coleoptera) “COI-delimitation” is widely accepted. This study suggests that strict division of gammarids into species according to COI can lead to overestimation of species number (BIN analysis indicated the presence of up to 21 species only within *G. tatrensis*). In the case of more intensive use of molecular features in the delimitation and description of new taxa, a broader discussion will certainly be needed to provide generally acceptable rules.

## Conclusions

Molecular diversity of the studied *G. balcanicus* complex revealed the existence of several lineages that survived Pleistocene glaciations in local periglacial refugia in the northern Carpathians. These lineages show a complex pattern of survival and elevated diversification through the Pleistocene, suggesting that the Ice age was a driving force of speciation. We observed two schemes of molecular diversity spatial patterns: firstly, lineages showing low molecular diversity over its geographic range, including the recently colonised areas, and, second, a pattern of numerous locally endemic lineages. All the described lineages can be aggregated in distinguishable MOTUs that represent putative separate species or cryptic and pseudo-cryptic species, whose existence is to be validated via integrative methods. In most cases, these putative cryptic species show postglacial population growth. We revealed a contrasting pattern of mitochondrial vs. nuclear diversification that probably is a result of the preservation of ancient polymorphism in extant lineages of the *G. balcanicus* complex. Additionally, we redescribed species *G. tatrensis*, described a new species *G. stasiuki* sp. nov. and improved barcode reference database by providing new data that can be used in future recognition and biodiversity assessment through molecular methods.

## Material and methods

### Material collection, identification and analysis

The amphipods were collected by kick-net sampling from 75 sites (BOLD: 10.5883/DS-GAMNCARP) representing the northernmost range of *G. balcanicus* and initially identified using available keys^8,41^. Individuals of *G. balcanicus* were selected for the following morphological and molecular analysis.

Selected sexually mature individuals of both sexes were dissected, and all appendages except of pleopods were stained with lignin pink (Azophloxin, C18H13N3Na2O8S2) and mounted with Euparal (Carl Roth GmBH, 7356.1) on microscope slides. Afterwards, they were photographed and drawn according to the protocol described by Coleman (2006, 2009). Twelve specimens were used for Scanning Electron Microscopy (SEM). SEM pictures were taken using dried specimens with 10 nm gold coating under Phenom ProX microscope in the Department of Invertebrate Zoology and Hydrobiology of the University of Lodz. Three magnifications were used 5000×, 10,000×, and 30,000×. Pictures were taken from two same-sized individuals from three molecularly distinct populations of *G. tatrensis* and one of *G. stasiuki* sp. nov. (see results).

### DNA processing and initial analysis

DNA was isolated from a total of 286 individuals. The isolation, amplification and sequencing followed the procedure from^20^. Altogether, five markers were amplified: two mitochondrial including the barcoding fragment of the cytochrome oxidase subunit I (COI, ca. 650 bp) and 16S ribosomal RNA (ca. 350 bp) as well as three nuclear markers: fragments of 28S ribosomal RNA (ca. 900 bp), histone H3 (ca. 300 bp) and elongation factor EF1-alpha (ca 500 bp). The set of primers used in the study is provided in Table S1. Obtained sequences were tested for contamination via BLASTN^65^ and were verified as Gammaridae. Sequences of each respective gene fragment were assembled, aligned and trimmed to the same length. Their alignment was performed using MAFFT with automatic settings to select the best algorithm for each data set^66^. All newly generated sequences were deposited in GenBank and BOLD dataset DS-GAMNCARP. The data set was supplemented by the already published sequences from^20,24^. Altogether, our data set consisted of 325 sequences for COI, 80 for 16S, 83 for 28S, 82 for H3 and 72 for EF1-alpha. Heterozygous sites, observed for the EF1-alpha and H3, were coded as ambiguous nucleotides according to IUPAC code. To assess a potential loss in the phylogenetic signal, each single-marker data set was tested for saturation of substitutions with DAMBE 5.3^67^ using the index proposed by^68^. In order to visualise distances between sequences, the phylogeny was reconstructed for each single-marker data sets in MEGA X^69^ using the Neighbor-Joining (NJ) method^70^ and Kimura 2-parameter (K2p) distance^71^ with 1,000 replicates^72^. Ambiguous sites and gaps were treated as complete deletions (Fig. S1). For COI, only sequences above 500 bp long were used. Additionally, NJ tree was reconstructed following the same procedure using COI haplotypes.

### Species delimitation methods

To identify the number of molecular operational taxonomic units (MOTUs) that could represent putative cryptic/pseudo-cryptic species within *G. balcanicus*, we applied eight different methods. Three were distance-based: (1) Barcode Index Number (BIN) System^73^ , (2) barcode-gap approach using the Automatic Barcode Gap Discovery (ABGD) software^74^ and ASAP: assemble species by automatic partitioning^28^. In ABGD we used primary partitions as a principal for group definition, as they are typically stable on a broader range of prior values, minimise the number of false-positive (over-split species) and are usually close to the number of taxa described by taxonomists^74^. Both in ABGD and ASAP t he standard K2p distance correction was applied. The default values of 0.001 to 0.1 were explored as intraspecific distances and in ABGD gap values from 1 to 1.5 were applied (already tested approach, e.g.,^22^). The remaining five methods were based on phylogeny reconstruction. As a proxy for phylogeny-based methods, Bayesian tree was reconstructed in BEAST 2.5.2^75^. The site model was set up with bModelTest^76^. The tree prior was set to Birth–Death following Bayes factors (> 2 to next model) selection through Path Sampling. Four runs of Markov chain Monte Carlo (MCMC) were performed each 20 M generations-long, sampled every 2000 generations. Runs were examined for convergence in Tracer 1.7^77^. All runs reached the effective sample size (ESS) above 200 and were combined using LogCombiner 2.5.2. The final tree was summarised with TreeAnnotator 2.5.2, all being part of BEAST 2.5.2 package. The outgroup (*G. balcanicus* from locus typicus) was removed in all species delimitation analysis. Two different general mixed Yule coalescent (GMYC) model-based approaches^78^ were applied, one using the (3) single threshold and the other one (sGMYC) (4) multiple-threshold model (mGMYC) are used to estimate the boundary between intra- and interspecific branching patterns. Both analyses were performed in R software package 'SPLITS' (Species Limits by Threshold Statistics)^79^ in R v3.1.0^80^. The presence of significant differences between the two models was tested using the likelihood ratio test (LRT) in R package 'spiderDEV'. (5) Bayesian implementation of the Poison Tree Processor (bPTP)^81^ was performed on the bPTP webserver (available at https://species.h-its.org) with 500,000 iterations of MCMC and 10% burn-in. (6) The multi-rate PTP (mPTP)^82^ implements MCMC sampling that provides a fast and comprehensive evaluation of the inferred delimitation. Five runs of 100 M MCMC generations-long chain with burn-in of 10% were performed on the local server. All the mentioned methods are based on a single marker only, COI in this case, therefore we also used the multimarker delimitation method (7) STACEY v.1.2.1 (species tree and classification estimation, yarely)^83^ in BEAST 2.5.2^76^. All markers were used in the analysis; each COI haplotype not represented by a nuclear marker was removed from the analysis. The minimal clusters were determined by BINs. The nucleotide substitution models were set up with the bModelTest. The Birth–Death model was used to estimate the species tree, other priors were set according to authors guidelines and initially tested (priors: Collapse Height = 0.001, Collapse Weight = 0.5 using a beta prior [1.1], bdc growth rate log-normal [4.6, 2] population prior scale in inverse gamma [2.2], the relative death rate [1.1]). Ploidy was equal to 2 for nuclear genes and 0.5 for mtDNA genes. Two runs were performed with 100 M generations of MCMC sampled every 10,000 generations. The delimitation results were analysed with speciesDA^84^ with different settings for the CollapseHeight parameter. Moreover, we tested other species delimitation hypotheses, based on BINs, ABGD, and morphological delimitation (Morphological form A and B) employing multispecies coalescent model: *BEAST package in BEAST 2.5.2. Species trees were constructed utilising each of the mentioned species hypotheses and using the same settings and data set as for the STACEY analysis. The marginal likelihood of each hypothesis was calculated using path sampling in BEAST 2.5.2 with 25 steps and 10 M MCMC generations. Bayes factors were calculated for each hypothesis.

### Time calibrated reconstruction of phylogeny and speciation rate through time

For estimating and visualising the temporal framework of *G. balcanicus* and its sister lineages, we reconstructed the time-calibrated species tree using COI haplotypes and all available markers as separate partitions in the *BEAST package of BEAST 2.5.2^75^. The provisional “species” were defined as a priori according to the BINs provided by BOLD. The molecular clock was calibrated using the COI rate of 0.0166 substitutions/site Ma^−1^ (SD: 0.0022). The value is an average of rates from two independent studies focused on *G. balcanicus* morphospecies:^20^ (0.0167 Ma^−1^, SD: 0.0026) and^24^ (0.0165 Ma^−1^, SD: 0.0018). The material and strategies to calibrate the molecular clock with known geological events were different but resulted in almost identical rates. Additionally, these calibration schemes were cross-validated in the mentioned studies. The ploidy model was set according to the marker type (mtDNA vs nDNA) and the substitution model was selected via bModelTest. Four runs of the MCMC, each 20 M generations long and sampled every 2,000 generations. Results were processed the same way as in case of phylogeny reconstruction for species delimitation. To explore and visualise putative changes of speciation rates through time, and to interpret them in a spatiotemporal context, we have performed two analyses. First, the history of diversification was visualised as a lineage through time (LTT) plot generated in Tracer 1.7.1 from a subset of 1,500 Bayesian chronograms for the species trees generated by *BEAST. The subset of trees was generated using LogCombiner 2.5. Second, we modelled the macroevolutionary dynamics of diversification across the phylogeny with the program Bayesian Analysis of Macroevolutionary Mixtures—BAMM^85^. As input, we used the maximum clade credibility species chronogram generated in *BEAST. First, the priors were preselected using the R package 'BAMMtools'^86^. Four chains of MCMC were used, each 10 M generations long and sampled and chain swap proposed every 1,000 generations. The ESS was checked using R package 'coda' (Plummer et al. 2006) and proved to be > 200. Additionally, visual inspection of MCMC confirmed convergence. Post-run analysis and visualisation were performed using the R package 'BAMMtools'.

### Demographic analysis and haplotype network visualisation

The historical demographic patterns were explored using the COI data employing two approaches. First, to test for a recent demographic expansion, Tajima’s D, Fu’s Fs and Ramos-Onsins and Rozas R2 (Ramos-Onsins & Rozas 2002) indices were calculated using DNAsp6 software^87^. Their statistical significance was evaluated using coalescent simulations with 1,000 replications. Second, the extended Bayesian skyline plot (eBSP)^88^ in BEAST 2.5.2 was used to visualise demographic changes through time. The clock model, rate and priors on substitution models for each group were determined in the same way as for the time-calibrated phylogeny, and the population model was set to 0.5. Two MCMC chains were run to ensure convergence for 40 M iterations, sampled every 20,000 iterations, or both values were doubled to provide good ESS values (> 200). One run for each data set was used to plot the eBSP in an R script^80^ after a 10% burn-in phase. All demographic analyses were done also for ABGD delimited MOTU’s with a sufficient number of samples available. To visualise the haplotype and MOTUs relationships for nuclear markers, haplotype networks were reconstructed. Relationships between haplotypes of 28S (excluding outgroups) were reconstructed using median-joining (MJ) network in POPART 1.7^89^. The homoplasy level parameter (ε) was set at the default value (ε = 0). Relationships for EF1-alpha and H3 nuclear markers, that show the presence of heterozygosity, were reconstructed using the haploweb approach^90^. This method allows showing additional connections between haplotypes found co-occurring in heterozygous individuals. All sequences containing ambiguous sites coded with IUPAC code were phased using the software PHASE^91^ according to author guidelines. The full EF1-alpha and H3 data sets, excluding outgroups, were used to generate a network through the Median Joining algorithm using the HaplowebMaker tool^92^.

### Ethics approval and consent to participate

All applicable international, national and institutional guidelines for the care and use of animals were followed. All procedures performed in studies involving animals were in accordance with the ethical standards of the institution at which the studies were conducted.

The material was collected in accordance with the permits issued by The District Office, Department of Environmental Care Trenčín (OU-TN-OSZP1-2015/001937-12/Du) and the Ministry of the Environment of the Slovak Republic (5198/2015-2.3, 3735/2015-2.3).

## Supplementary Information

Supplementary Figure S1.

Supplementary Figure S2.

Supplementary Figure S3.

Supplementary Figure S4.

Supplementary Tables.

## Data Availability

GenBank accession numbers: COI-5P: OK502255 - OK502553, 16S: OK504316 - OK504395, 28S: OK504400 - OK504482, EF1-alpha: OK505720 - OK505791, H3: OK623835 - OK623916. All metadata and sequences are stored in BOLD Dataset DS-GAMNCARP (10.5883/DS-GAMNCARP). Type and paratype as well as DNA isolates are deposited in Museum and Institute of Zoology Polish Academy of Sciences: *G. stasiuki*: MIZ PAN CRU 1-26, *G. tatrensis*: MIZ PAN CRU 27-57.
