# Supplementary material for: Fuzzy species borders of glacial survivalists in the Carpathian biodiversity hotspot revealed using a multimarker approach
Source: Sci Rep. 2021 Nov 3;11:21629. doi: 10.1038/s41598-021-00320-8 (PMC8566499; doi:10.1038/s41598-021-00320-8)
Supplement: Supplementary file 1 — Supplementary Figure S1. [file 41598_2021_320_MOESM1_ESM.pdf]

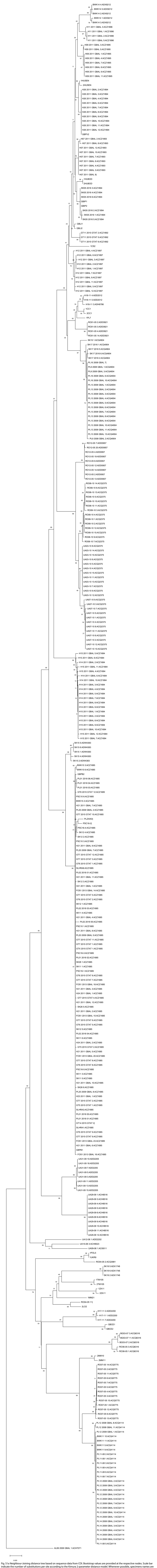

Fig. S1a Neighbour-Joining distance tree based on sequence data from COI. Bootstrap values are provided at the respective nodes. Scale bar indicate the number of substitutions per site according to the Kimura 2-parameter distance model. Whenever possible, specimens names are followed by BOLD BINs (see DS-GAMNCARP).

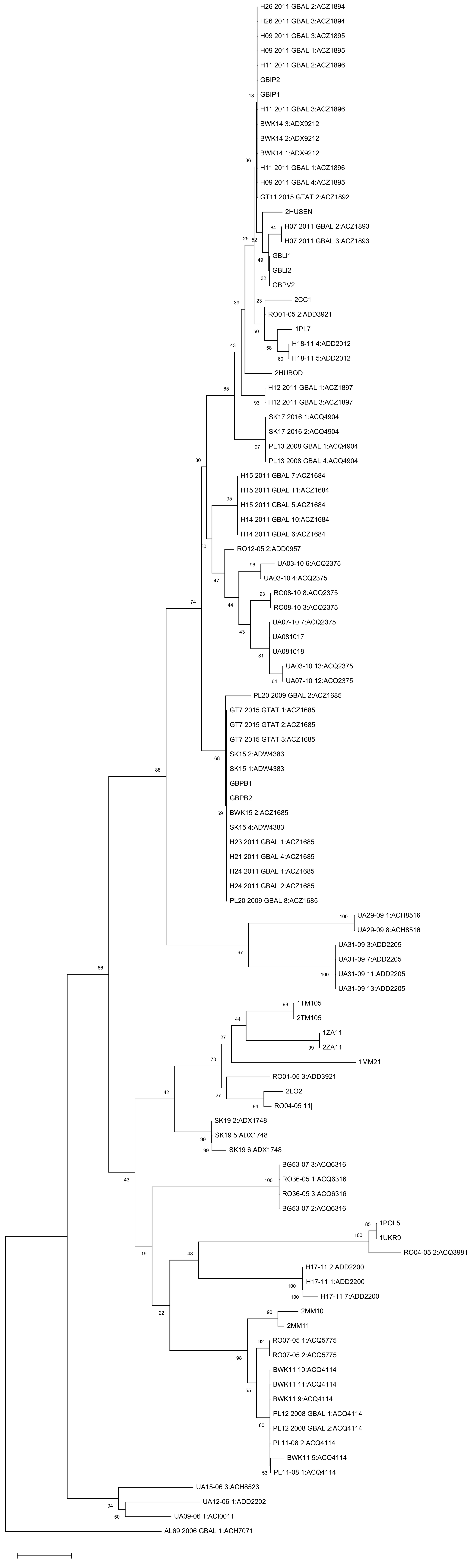

Fig. S1b Neighbour-Joining distance tree based on sequence data from 16S. Bootstrap values are provided at the respective nodes. Scale bar indicate the number of substitutions per site according to the Kimura 2-parameter distance model. Whenever possible, specimens names are followed by BOLD BINs (see DS-GAMNCARP).

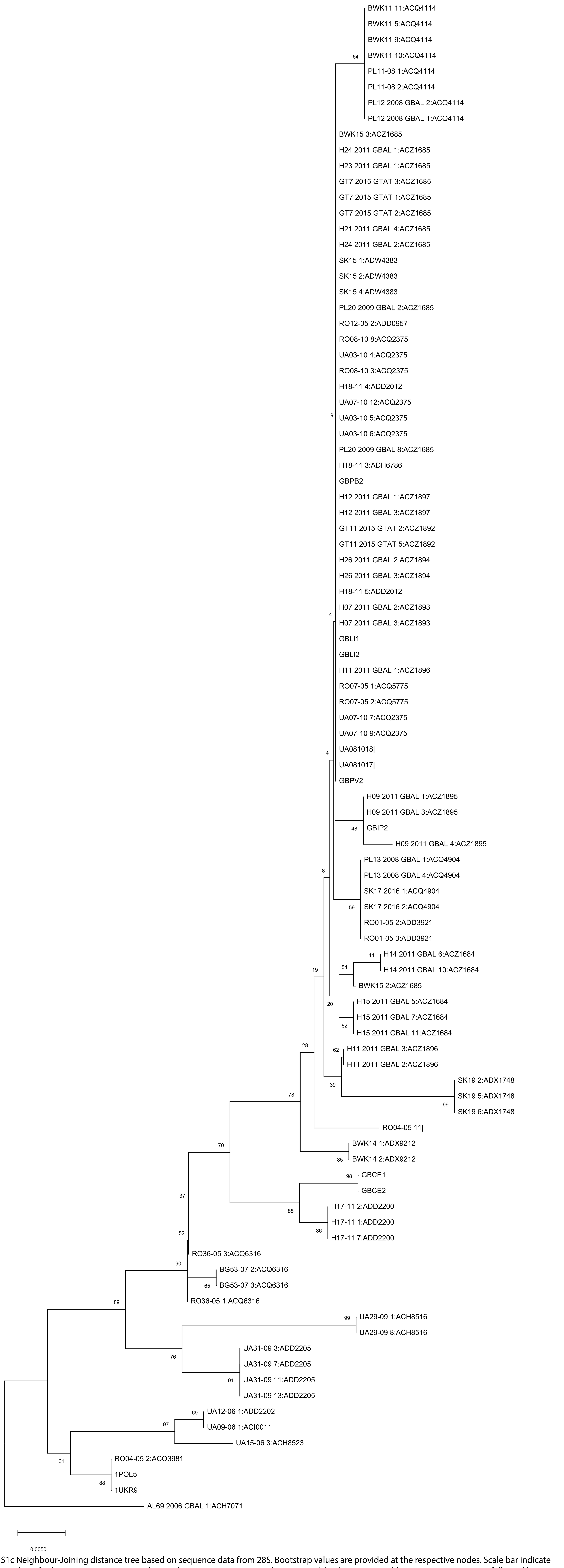

Fig. S1c Neighbour-Joining distance tree based on sequence data from 28S. Bootstrap values are provided at the respective nodes. Scale bar indicate the number of substitutions per site according to the Kimura 2-parameter distance model. Whenever possible, specimens names are followed by BOLD BINs (see DS-GAMNCARP).

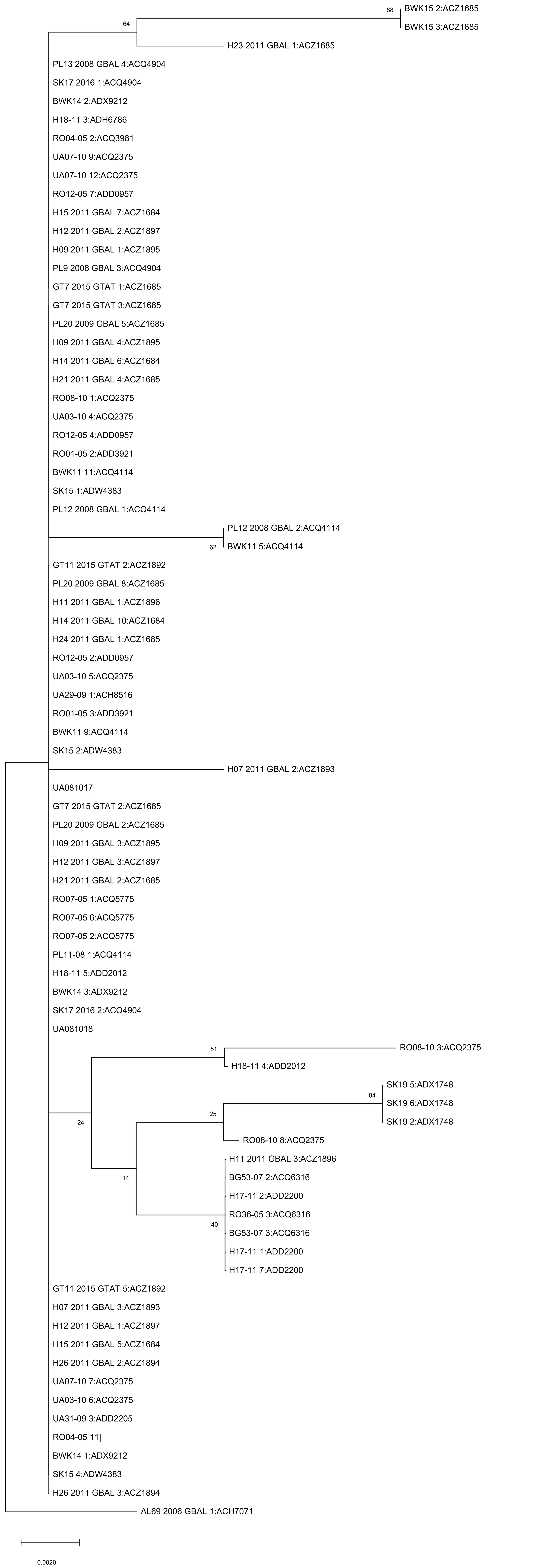

Fig. S1d Neighbour-Joining distance tree based on sequence data from H3. Bootstrap values are provided at the respective nodes. Scale bar indicate the number of substitutions per site according to the Kimura 2-parameter distance model. Whenever possible, specimens names are followed by BOLD BINs (see DS-GAMNCARP).

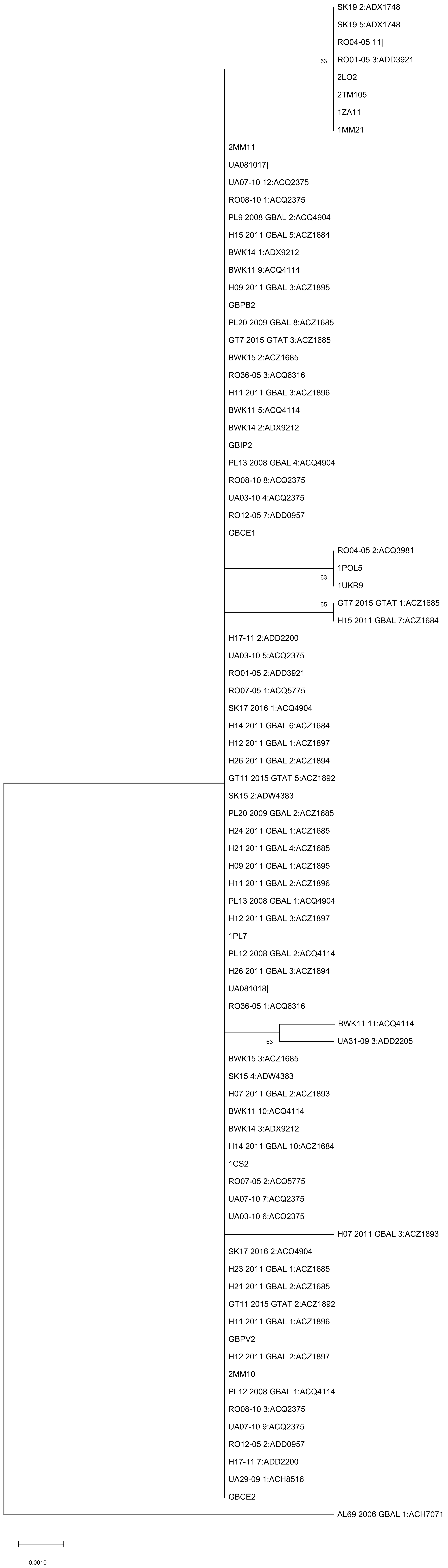

Fig. S1e Neighbour-Joining distance tree based on sequence data from EFa1. Bootstrap values are provided at the respective nodes. Scale bar indicate the number of substitutions per site according to the Kimura 2-parameter distance model. Whenever possible, specimens names are followed by BOLD BINs (see DS-GAMNCARP).
