# Supplementary figures and images for: Fuzzy species borders of glacial survivalists in the Carpathian biodiversity hotspot revealed using a multimarker approach
Source: Sci Rep. 2021 Nov 3;11:21629. doi: 10.1038/s41598-021-00320-8 (PMC8566499; doi:10.1038/s41598-021-00320-8)

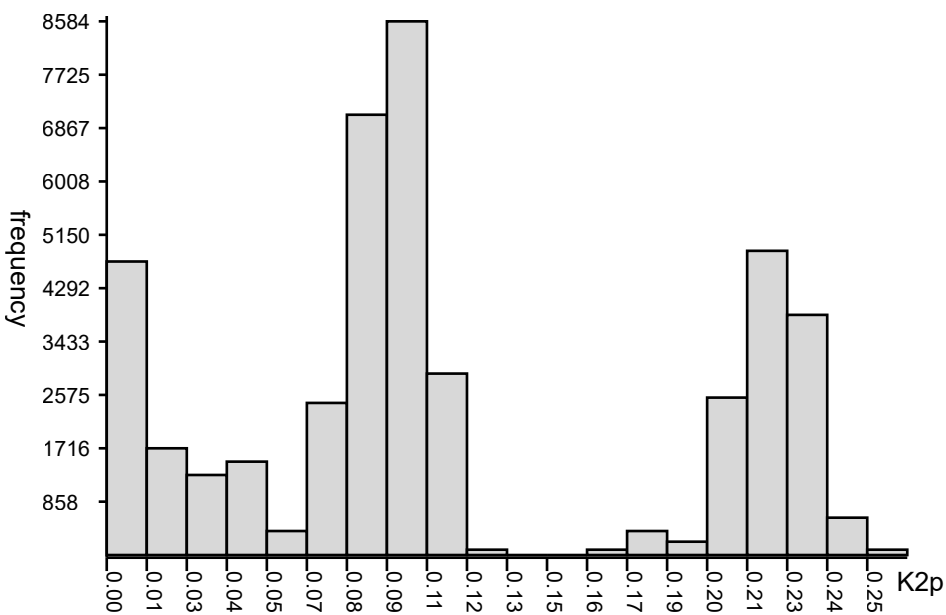

Fig. S2 The gap analysis. Distribution of pairwise k2distances obtained from ABGD

Supplement: Supplementary file 2 — Supplementary Figure S2. [file 41598_2021_320_MOESM2_ESM.pdf]

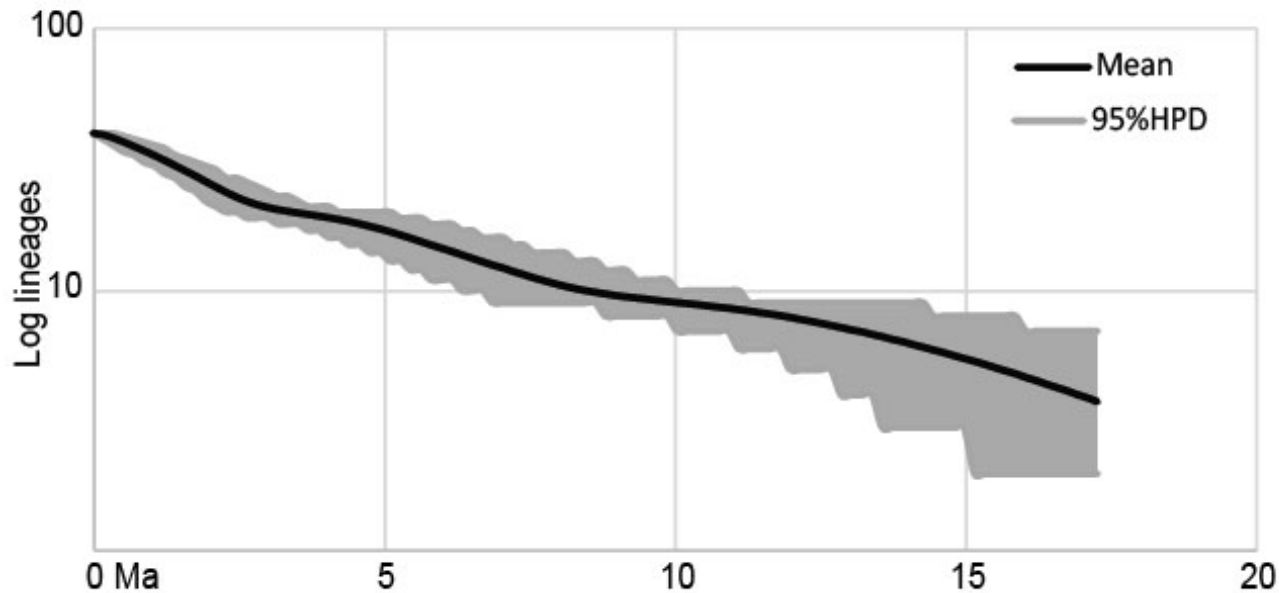

Fig. S3 Lineages through time plot obtained from Bayesian analysis (see Material and Methods)

Supplement: Supplementary file 3 — Supplementary Figure S3. [file 41598_2021_320_MOESM3_ESM.pdf]
