# Supplementary material for: Fuzzy species borders of glacial survivalists in the Carpathian biodiversity hotspot revealed using a multimarker approach
Source: Sci Rep. 2021 Nov 3;11:21629. doi: 10.1038/s41598-021-00320-8 (PMC8566499; doi:10.1038/s41598-021-00320-8)
Supplement: Supplementary file 4 — Supplementary Figure S4. [file 41598_2021_320_MOESM4_ESM.pdf]

Gammarus tatrensis typical Slovakia MOTU G1

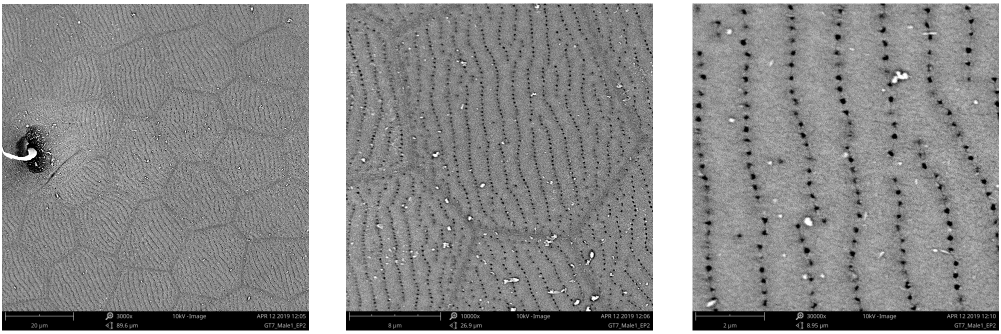

Gammarus tatrensis Poland MOTU G1

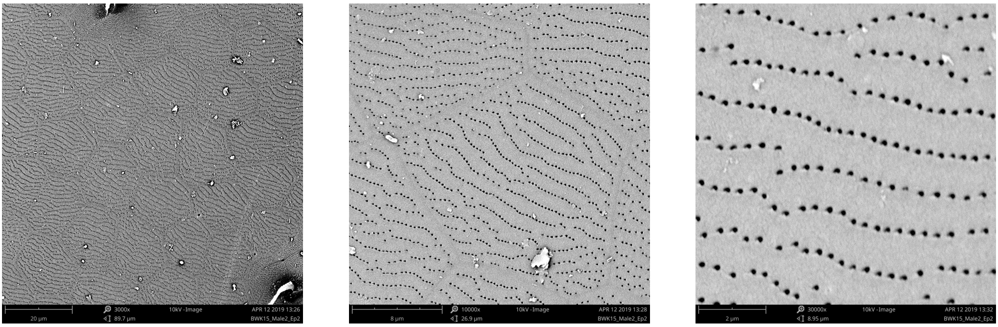

Gammarus tatrensis Hungary MOTU G4

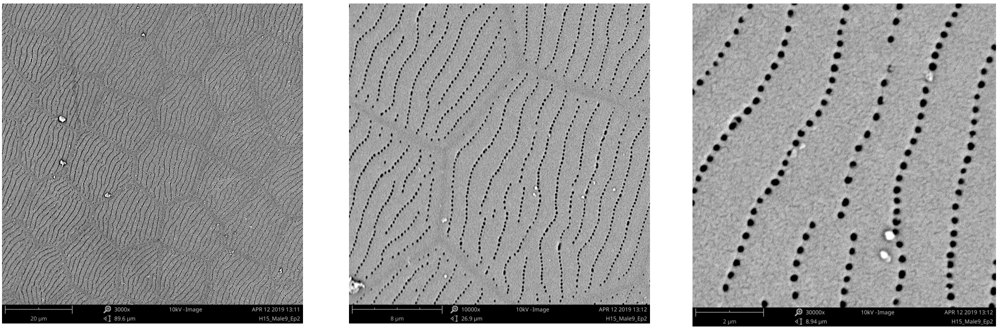

Gammarus stasiuki sp. nov

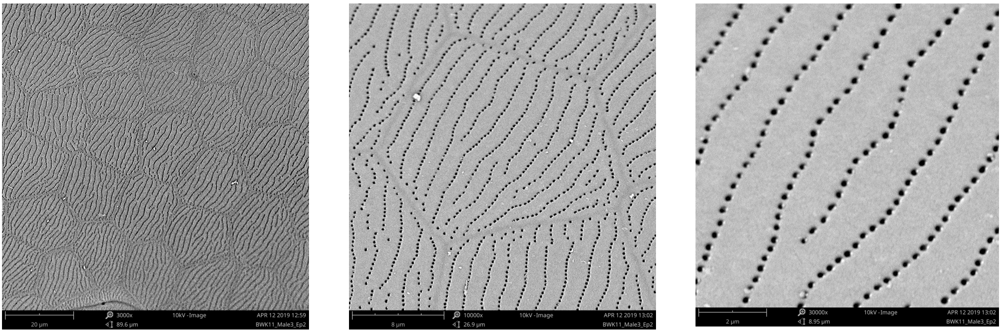

Fig. S4 Images of ultrastructure through SEM showing epimeral plate 2 of selected MOTUs. Magnification in each column from the left: 3000x, 10000x, 30000x.
