# Supplementary material for: Fuzzy species borders of glacial survivalists in the Carpathian biodiversity hotspot revealed using a multimarker approach
Source: Sci Rep. 2021 Nov 3;11:21629. doi: 10.1038/s41598-021-00320-8 (PMC8566499; doi:10.1038/s41598-021-00320-8)
Supplement: Supplementary file 5 — Supplementary Tables. [file 41598_2021_320_MOESM5_ESM.docx]

Table S1 List of primers used for amplification and sequencing.

| Gene | Primer | Sequence 5'-3' | Reference |
| --- | --- | --- | --- |
| COI | LCO1490 | GGTCAACAAATCATAAAGATATTGG | Folmer et al. (1994) |
|  | HCO2198 | TAAACTTCAGGGTGACCAAAAAATCA | Folmer et al. (1994) |
|  | UCOIF | TAWACTTCDGGRTGRCCRAAAAAYCA | Costa et al. (2007) |
|  | UCOIR | ACWAAYCAYAAAGAYATYGG | Costa et al. (2007) |
| 28S | 28F | TTAGTAGGGGCGACCGAACAGGGAT | Hou et al. (2007) |
|  | 28R | GTCTTTCGCCCCTATGCCCAACTGA | Hou et al. (2007) |
|  | 28S-1000R | GACCGATGGGCTTGGACTTTACACC | Hou et al. (2007) |
| 16S | LR-J-Gam | ATTTTAATTCAACATCGAGGTTGC | Müller et al. (2002) |
|  | LR-N-Gam | TTTAACGGCTGCGGTATTTTGAC | Müller et al. (2002) |
|  | 16STf | CCGGTTTGAACTCAGATCATGT | Palumbi et al. (1991) |
|  | 16SBr | GGTAWHYTRACYGTGCTAAG | MacDonald et al. (2005) |
| EFa1 | EFa1F | CACTACTGGTCATCTCATCTAC | Hou et al. (2011) |
|  | EFa1R | ACTTCCAGGAGAGTCTCAAAC | Hou et al. (2011) |
| H3 | H3NF | ATGGCTCGTACCAAGCAGAC | Colgan et al. (2000) |
|  | H3NR | ATAGTCCTTGGGCATGATTGTTAC | Colgan et al. (2000) |

Folmer O., Black M., Hoeh W., Vrijenhoek R. 1994 DNA primers for amplification of mitochondrial cytochrome c oxidase subunit I from diverse metazoan invertebrates. *Molecular Marine Biology Biotechnology,* 3, 294–299.

Colgan D.J., Ponder W.F., Eggler P.E. 2000. Gastropod evolutionary rates and phylogenetic relationships assessed using partial 28S rDNA and histone H3 sequences. *Zoologica Scripta* 29: 29–63.

Costa F.O., Henzler C.M., Lunt D.H., Whiteley N.M., Rock J. 2009. Probing marine *Gammarus* (Amphipoda) taxonomy with DNA barcodes. *Systematics and Biodiversity,* ***7***(4), 365-379.

Cristescu M.E.A., Hebert P.D.N. 2005. The “Crustacean Seas” — an evolutionary perspective on the Ponto-Caspian peracarids. *Canadian Journal of Fisheries and Aquatic Sciences* 62:505–517.

Hou Z., Fu J., Li S. 2007. A molecular phylogeny of the genus *Gammarus* (Crustacea: Amphipoda) based on mitochondrial and nuclear gene sequences. *Molecular Phylogenetics and Evolution* **,** 596–611.

Hou Z., Sket B., Fiser C., & Li S. 2011. Eocene habitat shift from saline to freshwater promoted Tethyan amphipod diversification. Proceedings of the National *Academy of Sciences of the United States of America* 108(35):14533-14538.

MacDonald K.S., 3rd, Yampolsky L., & Duffy J.E. 2005. Molecular and morphological evolution of the amphipod radiation of Lake Baikal. *Molecular Phylogenetics and Evolution* 35(2):323-343.

Müller J.C., Schramm S., Seitz A. 2002. Genetic and morphological differentiation of *Dikerogammarus* invaders and their invasion history in Central Europe. *Freshwater Biology,* 47, 2039-2048.

Palumbi, S., A. Martin, S. Romano, W. Mcmillan, L. Stice & Grabowski G. 1991. The Simple Fool’s Guide to PCR. A Collection of PCR Protocols, Version 2. University of Hawaii, Honolulu.

Table S2 Marginal likelihoods estimates (MLE) from path sampling method for different species hypotheses.

| Species hypothesis | MLE 1 | MLE 2 | MLE 3 |
| --- | --- | --- | --- |
| BIN-Stacey | -13515.72246 | -13462.82779 | -13339.21655 |
| ABGD | -13640.24043 | -13627.36237 | -13619.86952 |
| Morphological | -13810.63501 | -13616.80317 | -13816.4696 |
